# Supplementary material for: CancerGD: A Resource for Identifying and Interpreting Genetic Dependencies in Cancer
Source: Cell Syst. 2017 Jul 26;5(1):82–86.e3. doi: 10.1016/j.cels.2017.06.002 (PMC5531859; doi:10.1016/j.cels.2017.06.002)
Supplement: Document S1. Figure S1, Table S1, and Methods S1 [file mmc1.pdf]

Cell Systems, Volume *5*

Supplemental Information

CancerGD: A Resource for Identifying and Interpreting Genetic Dependencies in Cancer

Stephen Bridgett, James Campbell, Christopher J. Lord, and Colm J. Ryan

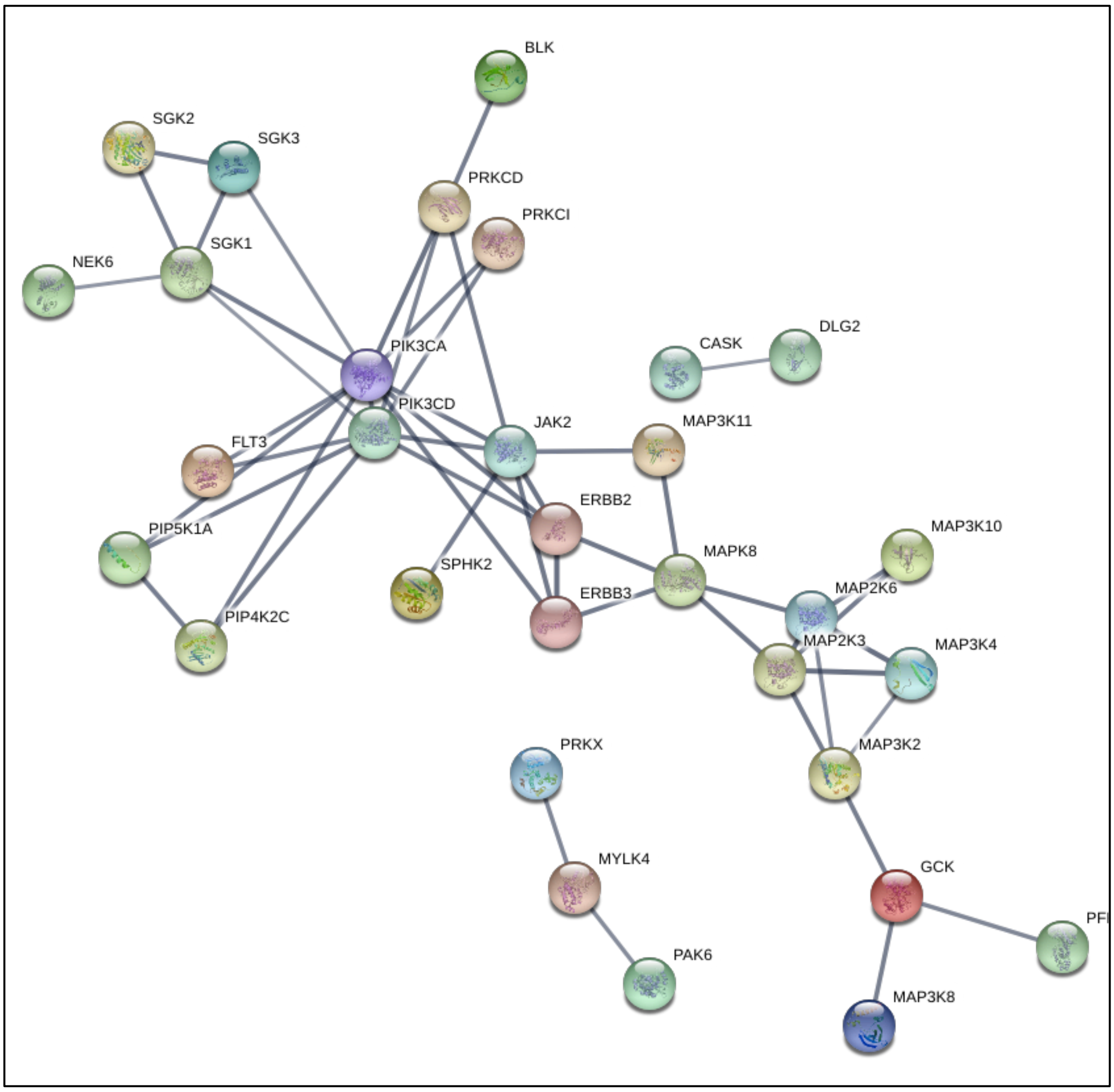

Figure S1. Visualizing the known interactions between all CGDs associated with a specific driver gene. Related to Figure 2.

High confidence STRING functional interactions between CGDs associated with *ERBB2* amplification in Campbell *et al* are shown.

Table S1. Driver genes currently included in CancerGD. Related to Figure 1

Gene	Studies	Tissues	CGDS
ACVR2A	1	2	676
AFDN	1	1	396
AFF4	1	1	268
APC	1	2	905
ARID1A	4	5	2336
ARID1B	2	2	273
ASXL1	2	2	1648
B2M	1	1	294
BCOR	1	2	389
BRAF	1	2	562
BRCA1	1	1	798
BRCA2	2	3	1685
CCND1	3	3	1264
CCNE1	1	1	930
CDH1	3	2	1761
CDKN2A	5	8	4074
CDKN2C	1	3	1148
CTNNB1	1	2	455
EGFR	2	2	460
EP300	2	3	1698
ERBB2	4	3	2523
EZH2	1	1	587
FANCA	1	2	707
FBXW7	1	2	440
GNAS	2	3	1434
HEY1	1	1	643
KDM6A	1	3	743
KRAS	3	4	2205
MAP2K4	2	2	1048
MDM2	1	1	224
MSH2	1	1	172
MSH6	1	2	651
MYC	3	5	1782
NCOA3	1	1	931
NCOR1	1	2	540
NF1	3	4	1271
NRAS	2	2	1232
PIK3CA	4	5	2800
PIK3R1	1	2	372
PPM1D	3	1	1156
PTCH1	1	1	197

PTEN	4	4	2176
PTPRK	1	1	289
RB1	3	3	1602
RNF43	2	3	1787
RPL22	1	2	836
SKP2	1	1	1095
SMAD4	3	4	1863
SMARCA4	2	2	437
SPOP	1	1	738
STK11	2	2	341
TP53	5	9	3690
UBR5	1	2	677

Methods S1. CancerGD short tutorial (20 mins)

Overview:

CancerGD.org provides a search interface for genetic dependencies identified in loss-of-function screens in panels of tumor cell lines. A genetic dependency is identified when there is a statistical association between the presence of a particular mutation and increased sensitivity to the inhibition of a specific gene. These dependencies are identified by integrating large-scale loss-of-function screens in panels of cell lines with genotype data for the same cell lines. In CancerGD we store all nominally significant dependencies ($P < 0.05$) with a common language effect size > 65% (see http://www.cancergd.org/faq/#effectsize for an explanation). A goal of this resource is to help understand genetic dependencies in the context of known functional interaction networks (e.g. protein-protein interactions). Towards this end we have developed simple functionality to identify those genetic dependencies that occur **within pathways** (i.e. where the driver gene and the target dependency belong to the same pathway) and **between pathways** (i.e. where the dependencies associated with a given driver gene belong to the same complex or pathway as each other). To further facilitate follow on studies we have also annotated all dependencies in the database according to the availability of inhibitors for the target genes.

Here we provide a simple tutorial that takes the user through the main functionality of www.cancergd.org. We show how CancerGD can be used to browse and analyse the dependencies associated with *ERBB2* amplification in the Campbell *et al* paper published in Cell Reports (2016). This tutorial should take approximately 20 minutes to complete.

Step 1 – retrieving the dependencies associated with a driver gene

Navigate to http://www.cancergd.org/ in your internet browser. You will see a search box resembling the below image. In the **Driver gene** field type *'ERBB2'*, in the **Tissue type** dropdown select *'Pan cancer'* and in the **Study** dropdown please select *'Campbell(2016)'*. Click the **Search** button

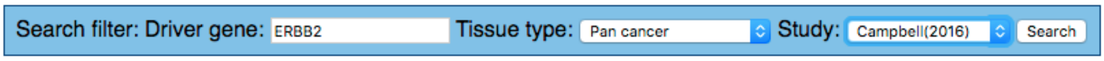

You will be presented with a table of results resembling the below image. The top of the page provides details (gene synonyms, a gene description, links to the gene on external resources) for the selected driver gene (*ERBB2)*. The bottom of the page is a table displaying all of the nominally significant dependencies associated with the selected driver gene (*ERBB2*) in the selected tissue (pan-cancer, i.e. across all tissue types) from the selected study (Campbell *et al*).

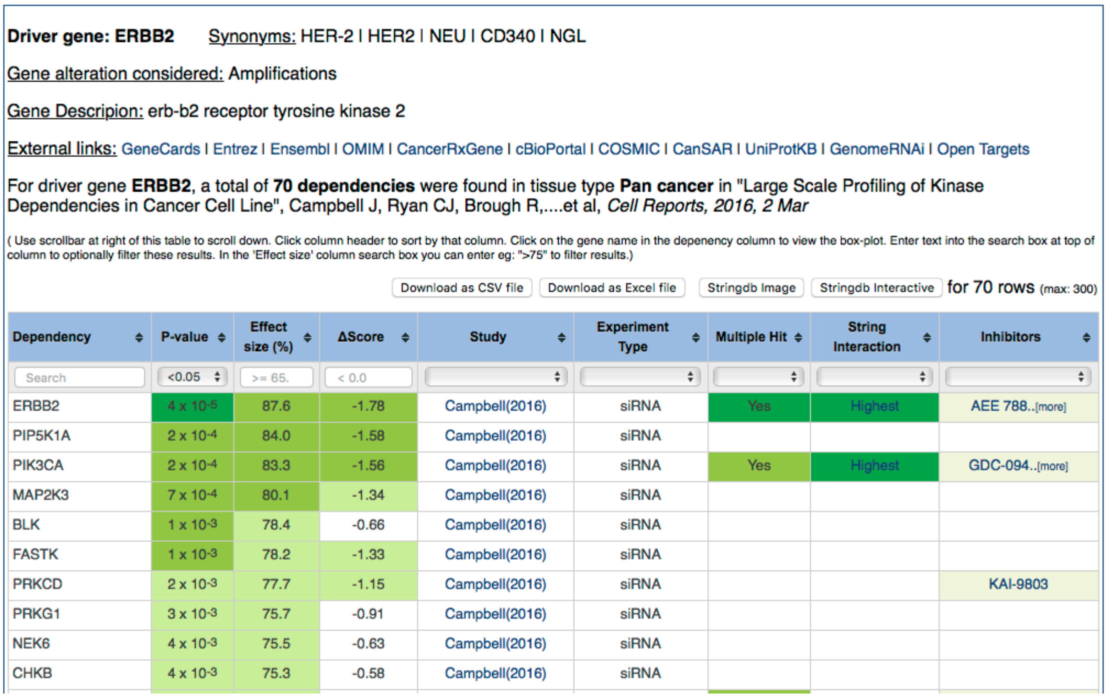

Driver gene: ERBB2 Synonyms: HER-2 I HER2 I NEU I CD340 I NGL

Gene alteration considered: Amplifications

Gene Descripion: erb-b2 receptor tyrosine kinase 2

External links: GeneCards I Entrez I Ensembl I OMIM I CancerRxGene I cBioPortal I COSMIC I CanSAR I UniProtKB I GenomeRNAi I Open Targets

For driver gene **ERBB2**, a total of **70 dependencies** were found in tissue type **Pan cancer** in "Large Scale Profiling of Kinase Dependencies in Cancer Cell Line", Campbell J, Ryan CJ, Brough R,....et al, *Cell Reports, 2016, 2 Mar*

(Use scrollbar at right of this table to scroll down. Click column header to sort by that column. Click on the gene name in the depenency column to view the box-plot. Enter text into the search box at top of column to optionally filter these results. In the 'Effect size' column search box you can enter eg: ">75" to filter results.)

Download as CSV file | Download as Excel file | Stringdb Image | Stringdb Interactive for 70 rows (max: 300)

Dependency	P-value	Effect size (%)	ΔScore	Study	Experiment Type	Multiple Hit	String Interaction	Inhibitors
Search	<0.05	>= 65.	< 0.0					
ERBB2	4×10^{-5}	87.6	-1.78	Campbell(2016)	siRNA	Yes	Highest	AEE 788..[more]
PIP5K1A	2×10^{-4}	84.0	-1.58	Campbell(2016)	siRNA			
PIK3CA	2×10^{-4}	83.3	-1.56	Campbell(2016)	siRNA	Yes	Highest	GDC-094..[more]
MAP2K3	7×10^{-4}	80.1	-1.34	Campbell(2016)	siRNA			
BLK	1×10^{-3}	78.4	-0.66	Campbell(2016)	siRNA			
FASTK	1×10^{-3}	78.2	-1.33	Campbell(2016)	siRNA			
PRKCD	2×10^{-3}	77.7	-1.15	Campbell(2016)	siRNA			KAI-9803
PRKG1	3×10^{-3}	75.7	-0.91	Campbell(2016)	siRNA			
NEK6	4×10^{-3}	75.5	-0.63	Campbell(2016)	siRNA			
CHKB	4×10^{-3}	75.3	-0.58	Campbell(2016)	siRNA			

Clicking on any gene name in the 'Dependency' column will present the user with a view of the evidence supporting that dependency. Click on *MAP2K3* to proceed to the next step

Step 2 – viewing the data supporting individual dependencies

You will be presented with a window resembling the below image. This view presents the data supporting the association between *ERBB2* amplification and sensitivity to RNAi reagents targeting *MAP2K3*.

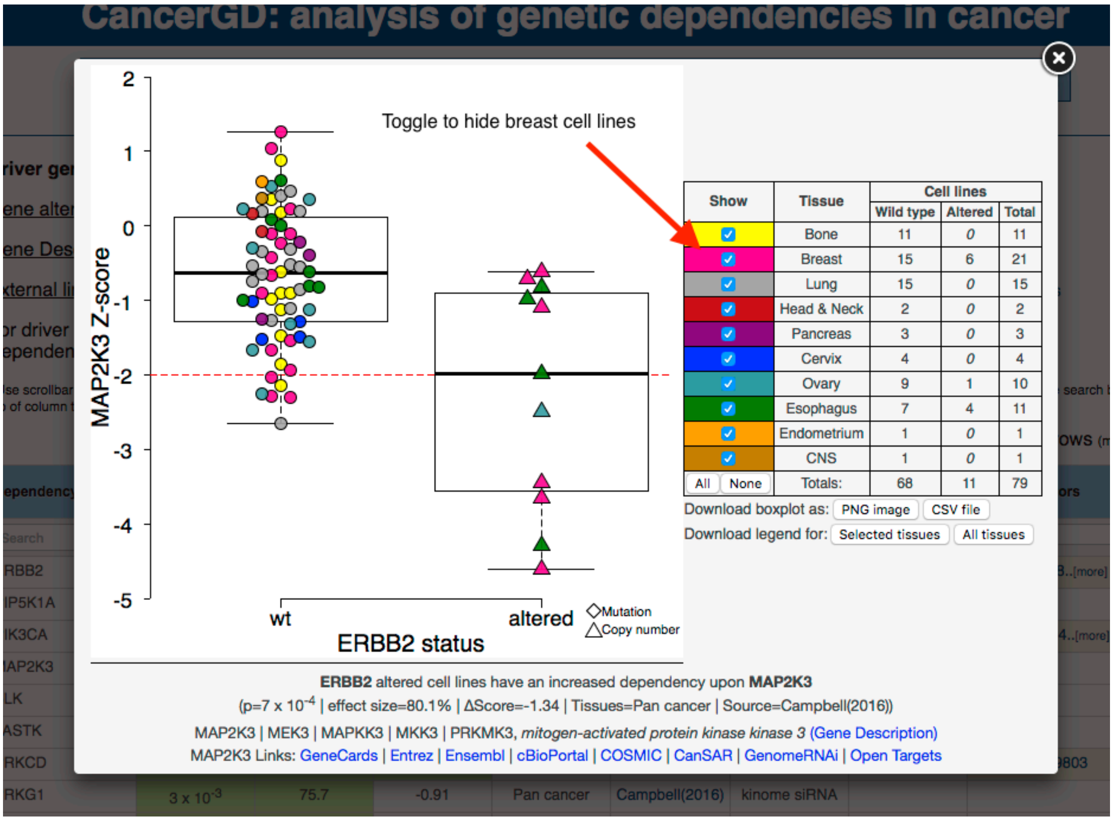

This is an interactive box plot (http://www.cancergd.org/faq/#boxplots) that displays the sensitivity of cell lines partitioned according to *ERBB2* status to RNAi reagents targeting *MAP2K3*. The cell lines featuring an alteration of *ERBB2* are displayed on the right and the cell lines without the alteration are on the left. Each colored shape represents a cell line and the position along the y-axis indicates how sensitive that cell line is to the RNAi reagents targeting the gene indicated (*MAP2K3)*. A lower position on the y-axis indicates greater sensitivity. The colors indicate the tissue of origin for each cell line, as indicated in the legend on the right hand side. Toggles in the legend facilitate hiding or displaying cell lines from specific histologies. To see how the dependency between *ERBB2* and *MAP2K3* appears when breast cell lines are removed uncheck the box beside ‘Breast’ in the legend.

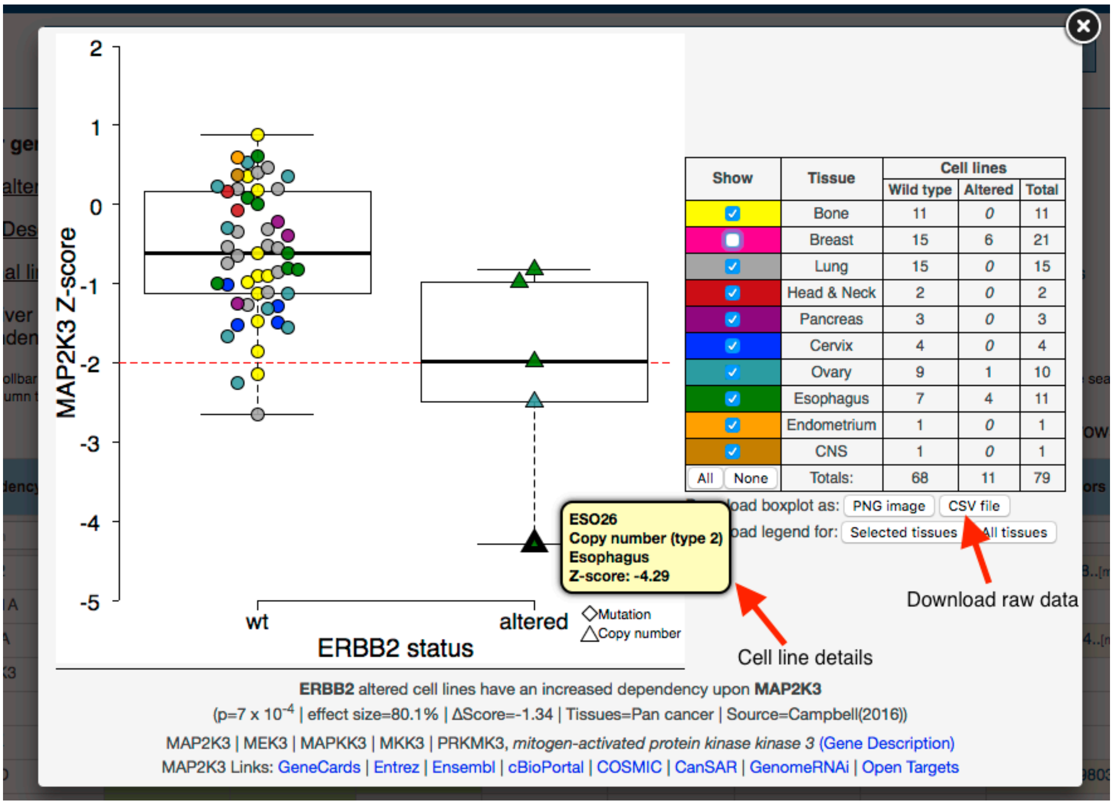

To download a high-resolution copy of this image click ‘Download boxplot as **PNG image**’. To download the raw data supporting this dependency in a comma separated text file, click ‘Download boxplot as **CSV file’**. This can be opened with Microsoft Excel or similar applications.

To see the details associated with a specific cell line hover your cursor over the shape corresponding to that cell line (e.g. above we hover over the cell line with the greatest sensitivity to *MAP2K3* inhibition).

Click the **X** in the top right to close this image and return to the table that lists genetic dependencies.

Step 3 – filtering dependencies with a known functional relationship to the driver gene

One of the goals of this resource is to facilitate the interpretation of genetic dependencies and to develop filters to prioritize promising candidates for follow up studies. The simplest approach is to focus on dependencies that have a known relationship (e.g. a protein-protein interaction) with the driver gene. To identify these - choose *'Any'* in the *'String Interaction'* column. This will filter the table to show only the genetic dependencies that have a functional relationship (e.g. protein-protein interaction) with *ERBB2* as displayed below.

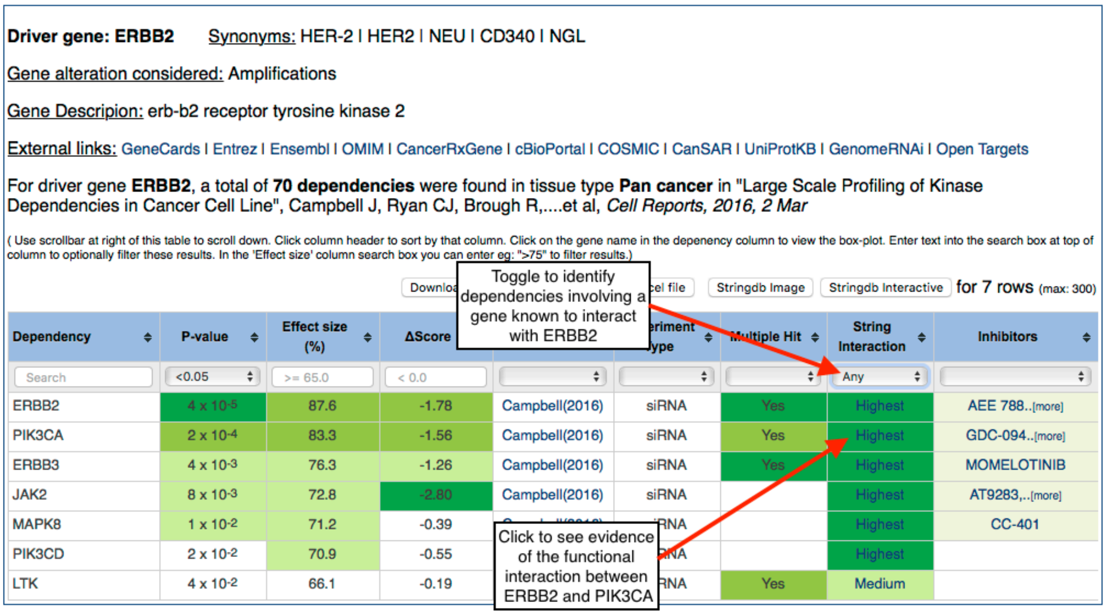

This identifies the *ERBB2* downstream effector *PIK3CA* and the *ERBB2* binding partner *ERBB3* among others. These functional relationships are obtained from the STRING database (http://string-db.org/). Clicking on text inside the *String Interaction* column (e.g. *Highest*) will bring the user to the STRING database where the data supporting the functional interaction between the driver gene and the dependency will be displayed.

Step 4 – identifying interactions between the dependencies associated with a driver gene

An alternative to identifying the known functional interactions between a driver gene and its dependencies is to try to understand the relationship between all of the dependencies associated with a given driver gene. In this way it may be possible to identify pathways or protein complexes upon which the driver gene is associated with an increased dependency. For this analysis we again rely on the STRING database (http://string-db.org/). To view all of the interactions between the dependencies associated *ERBB2* click on the **'Stringdb Image'** button above the dependencies table.

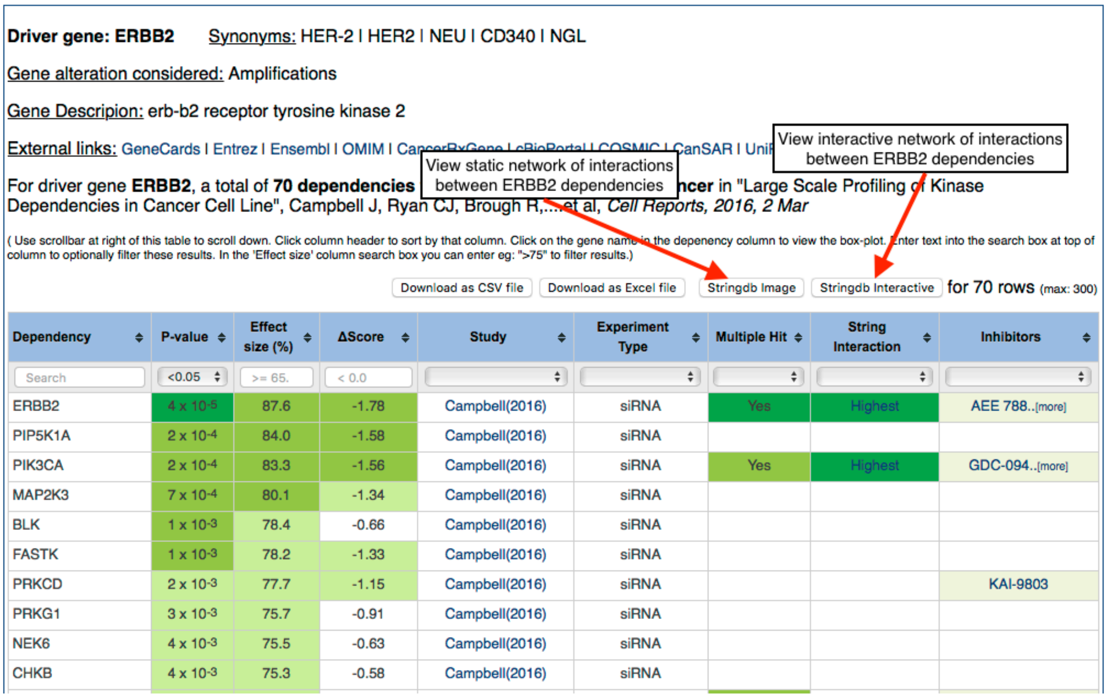

Driver gene: ERBB2 Synonyms: HER-2 | HER2 | NEU | CD340 | NGL

Gene alteration considered: Amplifications

Gene Descripion: erb-b2 receptor tyrosine kinase 2

External links: GeneCards | Entrez | Ensembl | OMIM | Ca[illegible] | CanSAR | Uni[illegible]

For driver gene **ERBB2**, a total of **70 dependencies** [illegible]**ncer** in "Large Scale Profiling of Kinase Dependencies in Cancer Cell Line", Campbell J, Ryan CJ, Brough R,...et al, *Cell Reports, 2016, 2 Mar*

(Use scrollbar at right of this table to scroll down. Click column header to sort by that column. Click on the gene name in the depenency column to view the box-plot. Enter text into the search box at top of column to optionally filter these results. In the 'Effect size' column search box you can enter eg: ">75" to filter results.)

Download as CSV file | Download as Excel file | Stringdb Image | Stringdb Interactive | for 70 rows (max: 300)

Dependency	P-value	Effect size (%)	ΔScore	Study	Experiment Type	Multiple Hit	String Interaction	Inhibitors
Search	<0.05	>= 65.	< 0.0					
ERBB2	4×10^{-5}	87.6	-1.78	Campbell(2016)	siRNA	Yes	Highest	AEE 788..[more]
PIP5K1A	2×10^{-4}	84.0	-1.58	Campbell(2016)	siRNA			
PIK3CA	2×10^{-4}	83.3	-1.56	Campbell(2016)	siRNA	Yes	Highest	GDC-094..[more]
MAP2K3	7×10^{-4}	80.1	-1.34	Campbell(2016)	siRNA			
BLK	1×10^{-3}	78.4	-0.66	Campbell(2016)	siRNA			
FASTK	1×10^{-3}	78.2	-1.33	Campbell(2016)	siRNA			
PRKCD	2×10^{-3}	77.7	-1.15	Campbell(2016)	siRNA			KAI-9803
PRKG1	3×10^{-3}	75.7	-0.91	Campbell(2016)	siRNA			
NEK6	4×10^{-3}	75.5	-0.63	Campbell(2016)	siRNA			
CHKB	4×10^{-3}	75.3	-0.58	Campbell(2016)	siRNA			

This will take a moment to retrieve an image similar to that below showing high-confidence functional interactions between the genes identified as *ERBB2* dependencies. You can see that *ERBB2* amplification is associated with an increased dependency upon a group of kinases functionally related to *ERBB2* and *PI3K* signaling, as well as a group of genes involved in map kinase signaling.

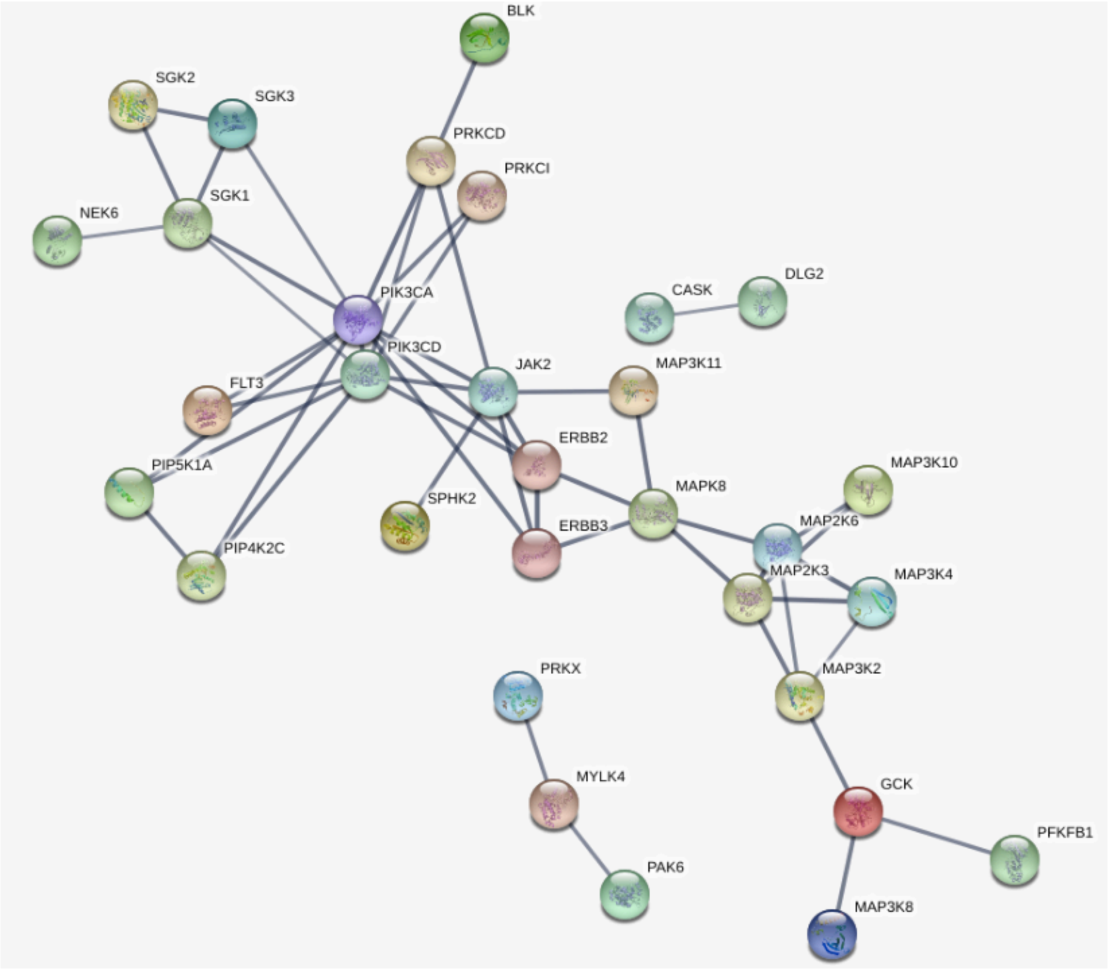

By selecting ‘**Stringdb Interactive**’ instead of **‘Stringdb image’** you can view an interactive version of this network on the STRING website. This will allow you to view the evidence supporting each functional interactions, to alter the layout of the network, and to filter the network in different ways. Click the **X** in the top right of the Stringdb image to close the image and return to the table listing dependencies.

Step 5 – identifying dependencies that can be exploited with existing inhibitors

A further goal of CancerGD is to facilitate follow on experimentation. One means to further explore or validate a dependency is to see if the same effect is observed using small molecule inhibitors rather than RNAi reagents. To that end we annotate all of our dependencies according to the availability of inhibitors. To view genes with available inhibitors, select '*Any*' in the '*Inhibitors*' column toggle. You will see a view resembling the below.

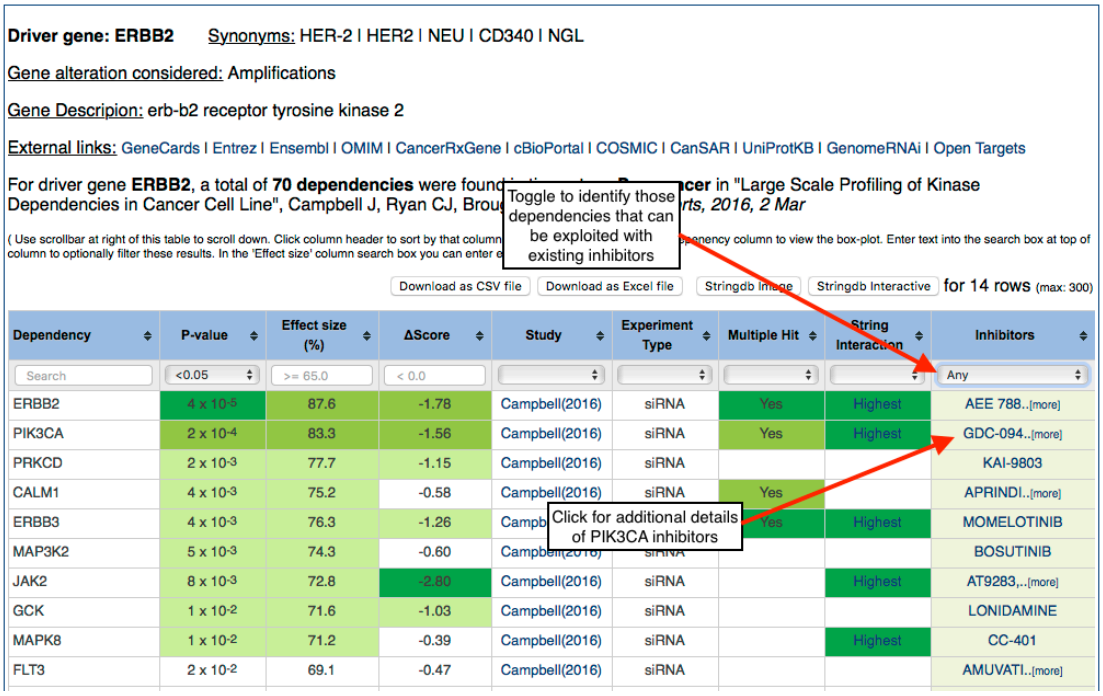

This filters the dependencies so that only those genes with known inhibitors are presented. The mapping from genes to inhibitors is taken from the DGIdb resource (http://dgidb.genome.wustl.edu/). Clicking on any inhibitor name in the *Inhibitors* column will bring the user to DGIdb, where details on the inhibitor are provided. For some genes there are more inhibitors available than can be presented in the *Inhibitors* column. These are indicated with the text *[more]*. Clicking on *[more]* in any entry in the *Inhibitors* column will display the full list of inhibitors associated with that gene in a window like that shown below :

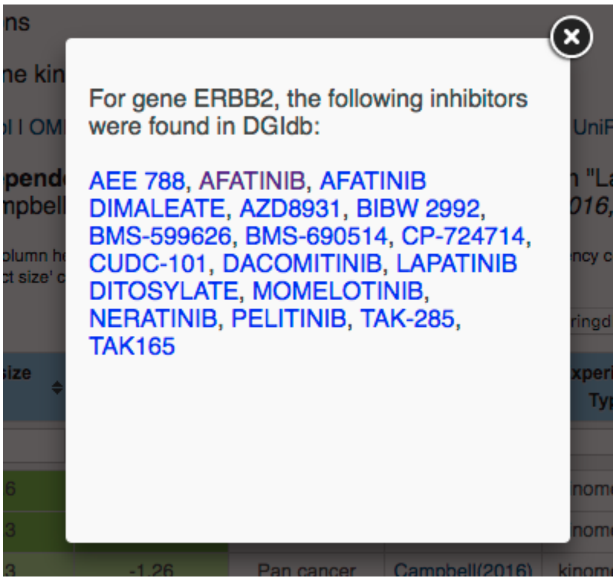

Clicking any inhibitor name within this window will bring the user to DGIdb, where details on the inhibitor are provided. Click the **X** to close this window.

Step 6 – identifying dependencies that have been observed in multiple datasets

A dependency observed in any one screen may be a statistical artefact, a context specific dependency, or a false positive resulting from the off-target effects of gene targetting reagents. Those dependencies observed in multiple independent datasets may make more promising candidates as they are less likely to be artefacts or false positive effects. To prioritise these for further validation, CancerGD allows easy filtering of the dependencies observed independently in multiple datasets. To view dependencies that have been associated with the same driver gene in the same tissue type, select '*Yes'* in the '*Multiple Hit'* column toggle. You will see a view resembling the below.

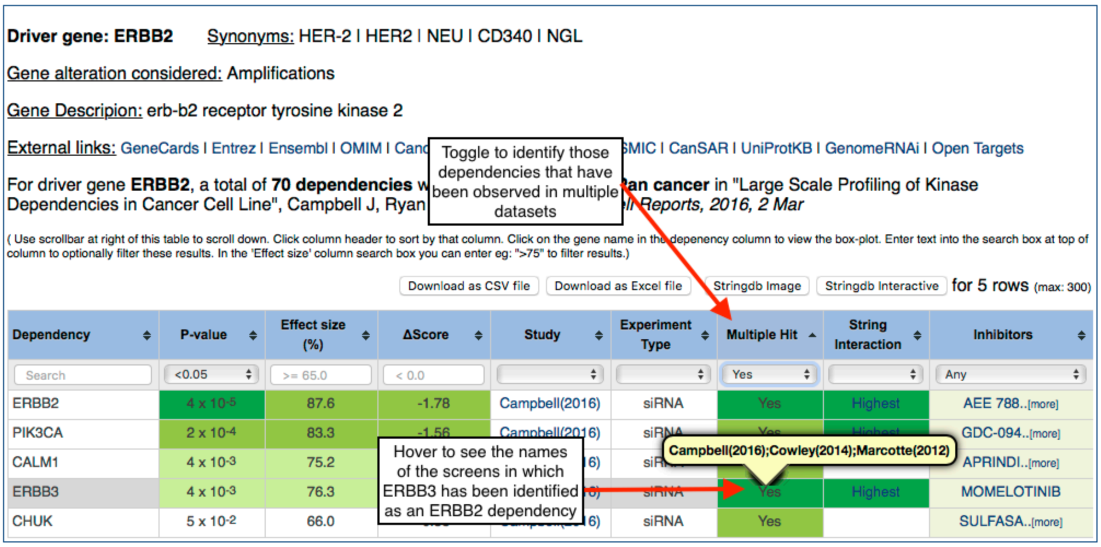

Hover over the *'Yes'* text in the "Multiple Hit" column to see the details of the screens that a specific gene has been identified as a dependency in.

Conclusion

You have now completed a tour of the main www.cancergd.org functionality. Further information is available on the FAQ (http://www.cancergd.org/faq/) page. We welcome feedback through the contact page (http://www.cancergd.org/contact/).
