# Supplementary material for: CancerGD: A Resource for Identifying and Interpreting Genetic Dependencies in Cancer
Source: Cell Syst. 2017 Jul 26;5(1):82–86.e3. doi: 10.1016/j.cels.2017.06.002 (PMC5531859; doi:10.1016/j.cels.2017.06.002)
Supplement: Document S2. Article plus Supplemental Information [file mmc2.pdf]

Tool

Cell Systems

CancerGD: A Resource for Identifying and Interpreting Genetic Dependencies in Cancer

Graphical Abstract

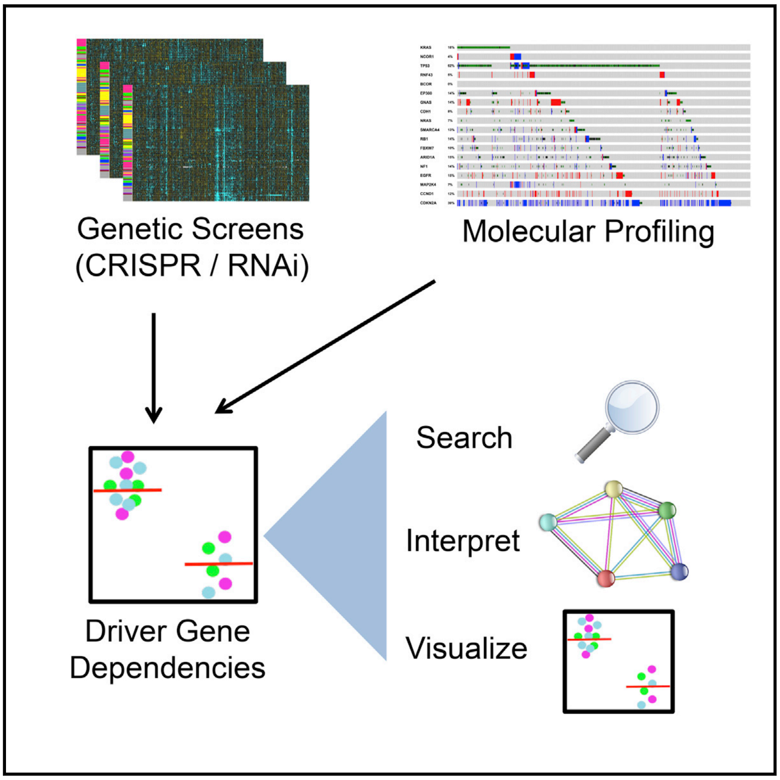

Authors

Stephen Bridgett, James Campbell, Christopher J. Lord, Colm J. Ryan

Correspondence

colm.ryan@ucd.ie

In Brief

We present a resource that integrates molecular profiling with loss-of-function screens to identify genetic dependencies in cancer cell lines.

Highlights

- Integrating loss-of-function screens with sequencing identifies genetic dependencies
- CancerGD facilitates searching and visualizing of dependencies from multiple sources
- CancerGD aids the interpretation of dependencies by integrating interaction networks

Bridgett et al., 2017, Cell Systems 5, 82–86
July 26, 2017 © 2017 The Authors. Published by Elsevier Inc.
http://dx.doi.org/10.1016/j.cels.2017.06.002

OPEN ACCESS
CellPress

Cell Systems
Tool

CancerGD: A Resource for Identifying and Interpreting Genetic Dependencies in Cancer

Stephen Bridgett,[1] James Campbell,[2] Christopher J. Lord,[2] and Colm J. Ryan[1,3,*]
[1]Systems Biology Ireland, University College Dublin, Belfield, Dublin, Ireland
[2]The CRUK Gene Function Laboratory and Breast Cancer Now Toby Robins Research Centre, The Institute of Cancer Research, London, UK
[3]Lead Contact
*Correspondence: colm.ryan@ucd.ie
http://dx.doi.org/10.1016/j.cels.2017.06.002

SUMMARY

Genes whose function is selectively essential in the presence of cancer-associated genetic aberrations represent promising targets for the development of precision therapeutics. Here, we present CancerGD, a resource that integrates genotypic profiling with large-scale loss-of-function genetic screens in tumor cell lines to identify such genetic dependencies. CancerGD provides tools for searching, visualizing, and interpreting these genetic dependencies through the integration of functional interaction networks. CancerGD includes different screen types (siRNA, shRNA, CRISPR), and we describe a simple format for submitting new datasets.

The ability to inhibit tumors in molecularly defined cohorts of patients is a cornerstone of precision cancer treatment. A successful approach has been the development of drugs that inhibit proteins specifically required in tumors harboring aberrations in recurrently altered cancer driver genes (Luo et al., 2009). For example, oncogene addiction effects, such as the increased sensitivity of *ERBB2* (*HER2*)-amplified breast tumors to ERBB2 inhibitors (Hynes and Lane, 2005), can be clinically exploited, as can non-oncogene addiction effects, such as the synthetic lethal relationship between *BRCA1*/*BRCA2* mutations and PARP inhibitors (Lord et al., 2015). To identify additional cancer genetic dependencies (CGDs) that may ultimately be exploited therapeutically, multiple groups have performed large-scale loss-of-function genetic screens in panels of tumor cell lines (Brough et al., 2011b; Campbell et al., 2016; Cheung et al., 2011; Cowley et al., 2014; Marcotte et al., 2012, 2016; Wang et al., 2017). Integrating the results of these screens with molecular profiling data creates hypothesis-generating resources where the hypotheses are of the form “tumor cells with a mutation in gene X are sensitive to inhibition of gene Y.” These hypotheses are typically tested in subsequent experiments—for example, in larger panels of cell lines, using orthogonal mechanisms of gene inhibition, and/or in mouse models—to ensure they are not statistical or experimental artifacts. Recent examples of novel CGDs discovered through genetic screening approaches include an increased sensitivity of *ARID1A* mutant cell lines to inhibition of the *ARID1A* paralog *ARID1B* (Helming et al., 2014), of *PTEN* mutant breast tumor cell lines to inhibition of the mitotic checkpoint kinase *TTK* (Brough et al., 2011b), and of *MYC*-amplified breast tumor cell lines to inhibition of multiple distinct splicing components (Hsu et al., 2015).

Although the results of loss-of-function screens are typically made publically available, their integration with genotypic data remains challenging for those without bioinformatics skills. Sequencing and copy-number data must be processed to identify likely functional alterations, cell line names matched between different data sources, and statistical analysis performed to identify associations between the alteration of driver genes and an increased sensitivity to inhibition of target genes. To address these challenges, we have developed CancerGD (www.cancergd.org), a resource that integrates multiple loss-of-function screens (Campbell et al., 2016; Cowley et al., 2014; Marcotte et al., 2012, 2016; Wang et al., 2017) with genotype data (Forbes et al., 2015; Iorio et al., 2016; Yang et al., 2013) to identify CGDs associated with a panel of cancer driver genes (Figure 1).

CancerGD currently facilitates the searching, visualization, and interpretation of CGDs (Figure 1) associated with 53 driver genes (Table S1). These genes were selected based on their identification as driver genes in multiple independent analyses (Campbell et al., 2016; Forbes et al., 2015; Vogelstein et al., 2013) and due to their alteration in at least three tumor cell lines featured in one or more of the included loss-of-function screens. Driver-gene-associated CGDs are identified both across cell lines from multiple histologies (Pan cancer) and within tumor cell lines arising from specific primary sites (e.g., Breast). With an intuitive search interface, it is thus possible to retrieve CGDs associated with *ERBB2* amplification across cell lines from all tissue types or specifically associated with *ERBB2* amplification in breast tumor models (Figure 2A). The data supporting every CGD can be visualized in an interactive boxplot (Figure 2B) and downloaded for reference.

Aside from oncogene addiction effects (Luo et al., 2009), which represent a tiny minority of the dependencies stored in CancerGD, the mechanistic interpretation of CGDs remains challenging. Why would mutation of one gene result in an increased dependency upon another? In yeast, the interpretation of such relationships has been greatly aided by the integration of protein-protein interaction networks with genetic screens (Kelley and Ideker, 2005). Following a similar model, to aid the interpretation of CGDs in CancerGD, we integrate functional interactions from the STRING database (Szklarczyk et al., 2015). This facilitates the rapid identification of CGDs involving gene pairs with known functional relationships. For instance, in the Campbell et al. (2016) dataset *ERBB2* amplification is associated with an increased dependency upon the *ERBB2* protein interaction

 © 2017 The Authors. Published by Elsevier Inc.
This is an open access article under the CC BY license (http://creativecommons.org/licenses/by/4.0/).
CrossMark

OPEN ACCESS
CellPress

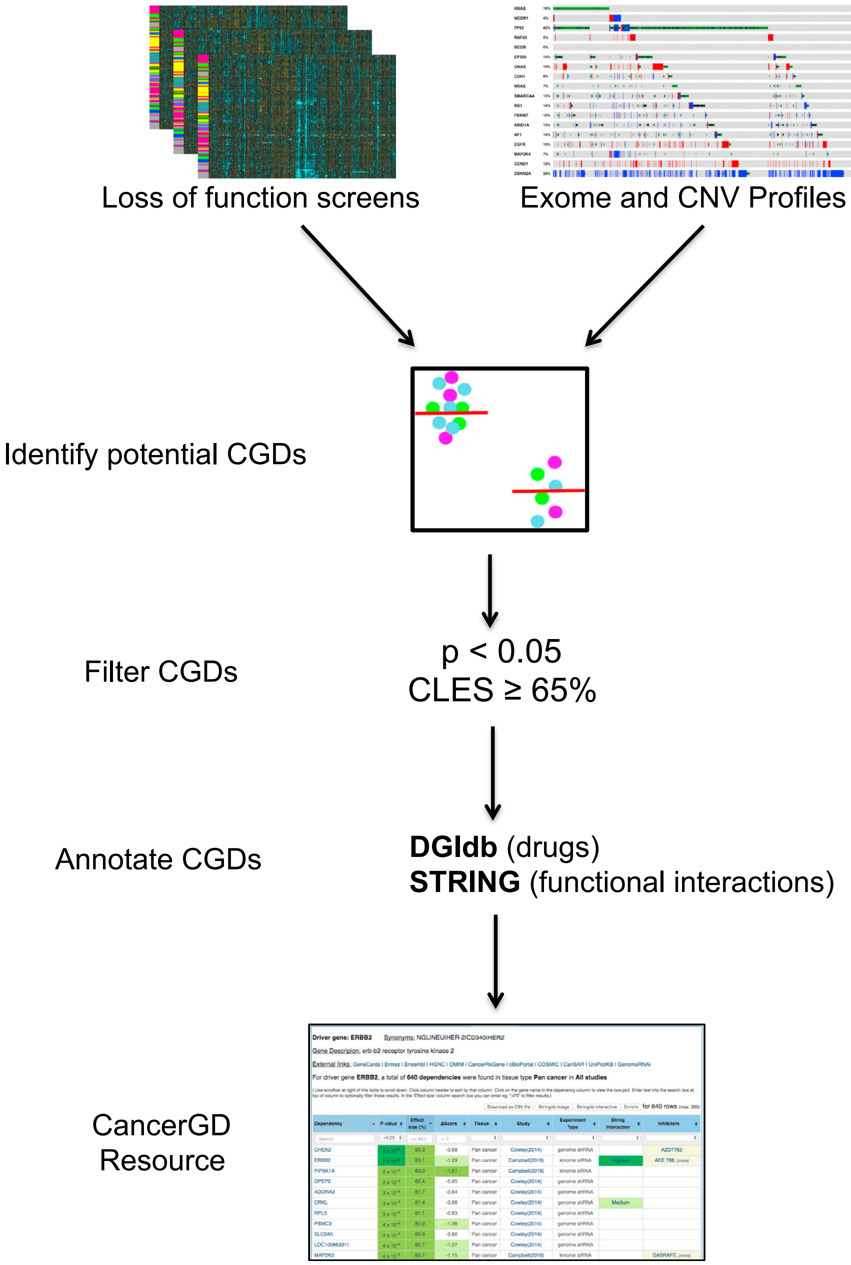

Figure 1. CancerGD Overview

Loss-of-function screens from multiple sources are integrated with exome and copy-number profiles from the GDSC resource. Cell lines are annotated according to the mutational status of a panel of driver genes (see Table S1). Statistical analysis is then performed to identify associations between the presence of driver gene alterations and sensitivity to reagents targeting specific genes. These CGDs are filtered such that only those with nominal significance ($p < 0.05$) and moderate common language effect sizes ($\geq 65\%$) are retained. Finally, all CGDs are annotated according to whether they occur between driver-target pairs with known functional relationships (STRING) and whether there is an inhibitor available for the target gene (DGIdb).

partners *JAK2* and *ERBB3*, as well as the *ERBB2* downstream effector *PIK3CA* (Figure 2A). Similarly in the Cowley et al. (2014) dataset loss or mutation of the BAF complex subunit *ARID1A* is associated with an increased dependency upon the *ARID1A* paralog and BAF complex member *ARID1B* (Helming et al., 2014). Such dependencies may make more promising candidates for follow-on experiments as they are supported by existing functional relationships in addition to the genetic association.

In addition to identifying known functional interactions between the driver gene and associated dependency, it can be helpful to understand the relationships between all of the CGDs associated with a given driver gene. For instance, we previously found that cell lines with a deletion or mutation of the tumor suppressor *SMAD4* display a strong dependency upon the mitotic checkpoint kinase *CHEK1* (Campbell et al., 2016). Considered in isolation, it is not clear whether this CGD relates to a specific function of *CHEK1* or a more general sensitivity to inhibition of the mitotic checkpoint. However, by analyzing all of the dependencies associated with *SMAD4*, we found that they were densely connected on the protein interaction network and primarily involved in the mitotic checkpoint (Campbell et al., 2016), suggesting a more general sensitivity to perturbation of this pathway. To facilitate the identification of such pathway-level dependencies, CancerGD provides network visualizations of the functional interactions between CGDs associated with each driver gene (Figure S1).

In contrast to the results of drug-screening efforts in panels of tumor cell lines (Barretina et al., 2012; Basu et al., 2013; Daemen et al., 2013; Garnett et al., 2012; Iorio et al., 2016; Yang et al., 2013), the CGDs identified in loss-of-function screens include targets that have no inhibitors available and consequently may serve as the rationale for the development of new small-molecule inhibitors. To facilitate the identification of CGDs that may be more readily exploited with available inhibitors, CancerGD integrates drug-gene interaction relationships from DGIdb (Griffith et al., 2013).

It has previously been highlighted that many CGDs identified in one loss-of-function screen are not evident in additional datasets (Brough et al., 2011a; Downward, 2015). This could indicate that these CGDs are context specific (Ashworth et al., 2011) but can also be explained by a variety of technical factors. Different screens feature different coverage of gene libraries (e.g., kinome versus genome-wide), different coverage of cancer types (e.g., only melanoma in one versus only breast in another), and different coverage of driver genes (e.g., many *BRAF* mutant

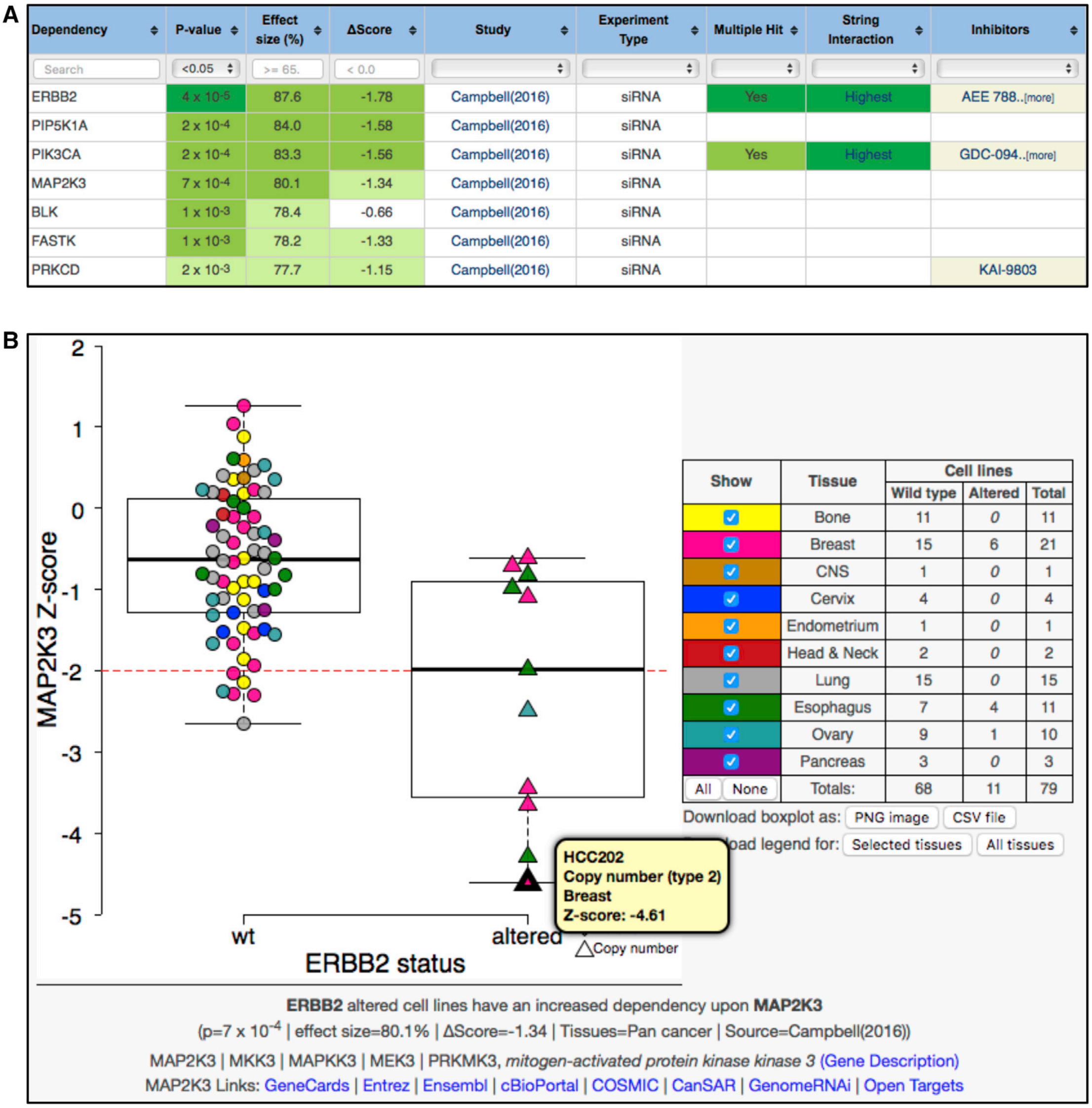

Dependency	P-value	Effect size (%)	ΔScore	Study	Experiment Type	Multiple Hit	String Interaction	Inhibitors
Search	<0.05	>= 65.	< 0.0					
ERBB2	4 x 10-5	87.6	-1.78	Campbell(2016)	siRNA	Yes	Highest	AEE 788..[more]
PIP5K1A	2 x 10-4	84.0	-1.58	Campbell(2016)	siRNA			
PIK3CA	2 x 10-4	83.3	-1.56	Campbell(2016)	siRNA	Yes	Highest	GDC-094..[more]
MAP2K3	7 x 10-4	80.1	-1.34	Campbell(2016)	siRNA			
BLK	1 x 10-3	78.4	-0.66	Campbell(2016)	siRNA			
FASTK	1 x 10-3	78.2	-1.33	Campbell(2016)	siRNA			
PRKCD	2 x 10-3	77.7	-1.15	Campbell(2016)	siRNA			KAI-9803

Figure 2. Genetic Dependency Exploration and Visualization

(A) The principle view of the database. Each row represents a gene identified as a dependency associated with *ERBB2* amplification in Campbell et al. (2016) across all tumor types (Pan cancer). Columns display experimental details along with the p value, common language effect size, and difference in median sensitivity score for each dependency. Genes identified as dependencies in multiple datasets are indicated in the Multiple Hit column. Genes with a known functional relationship to the driver gene (e.g., *PIK3CA*) are indicated in the String Interaction column, and drugs known to inhibit the target gene are indicated in the Inhibitors column. Toggles/search boxes permit easy filtering of interactions, e.g., to select only those genes with an associated inhibitor available. See also Figure S1 and the tutorial in Methods S1.

(B) Example boxplot showing an increased sensitivity of *ERBB2*-amplified cell lines to inhibition of *MAP2K3*. Each data point represents the sensitivity of a particular cell line to RNAi reagents targeting *MAP2K3*. Cell lines are grouped according to *ERBB2* amplification status with the wild-type group on the left and amplified group on the right. Cell lines are colored according to site of origin and toggles on the right permit the hiding/showing of cell lines from specific sites. Hovering over a given data point provides the cell line's name, the primary site, and the score associated with the RNAi reagent in that cell line. An overlapped box-whisker plot displays the interquartile range and the median for each group. High-resolution PNG images for each boxplot can be downloaded along with a CSV file containing all of the data presented in the boxplot. Links to the target gene (*MAP2K3*) on external websites are provided at the bottom of the plot.

cell lines in one screen versus none in another). These technical factors can result in the identification of CGDs in one screen that cannot be observed in a second screen. Furthermore, in any given screen there may be false positives resulting from the off-target effects of gene-targeting reagents (Jackson and Linsley, 2010) and false negatives resulting from variation in the knockdown efficiencies of different gene-targeting reagents (Kaelin, 2012). There are thus a number of explanations for why a CGD observed in one dataset may not be evident in another. Nonetheless, the CGDs that are observed in multiple datasets may be of particular interest as they are perhaps less likely to result from the off-target effects of gene-targeting reagents

and also less likely to be highly context specific. In CancerGD, we provide functionality to identify and filter those CGDs observed independently in multiple datasets.

CancerGD can incorporate datasets generated using different experimental and computational pipelines and is not restricted to loss-of-function screens generated using any specific method (shRNA/siRNA/CRISPR). The main requirement for inclusion is that a dataset must contain the results of screens in a panel of cell lines (a minimum of ten cell lines) and provide some quantitative measurement of the sensitivity of each cell line to the inhibition of each gene screened. Currently, the resource includes three genome-scale shRNA screens (Cowley et al., 2014; Marcotte et al., 2012, 2016), one kinome-wide siRNA screen (Campbell et al., 2016), and one genome-wide CRISPR screen (Wang et al., 2017). As additional screens become available, we will incorporate their results into the resource (see STAR Methods for instructions on how to format screens for easy inclusion in CancerGD).

A tutorial demonstrating the full functionality of CancerGD is provided in Supplemental Information. We believe that CancerGD will be a useful resource to aid a wider group of cancer researchers to benefit from the information generated in large-scale loss-of-function screens.

STAR★METHODS

Detailed methods are provided in the online version of this paper and include the following:

- KEY RESOURCES TABLE
- CONTACT FOR REAGENT AND RESOURCE SHARING
- METHOD DETAILS
  - Genotype Data
  - Loss of Function Screens
  - Cell Line Naming
  - Gene Identification
  - Drug Target Annotations
  - Functional Interactions
  - Implementation
  - Formatting Screens for CancerGD
- QUANTIFICATION AND STATISTICAL ANALYSIS
- DATA AND SOFTWARE AVAILABILITY

SUPPLEMENTAL INFORMATION

Supplemental Information includes one figure, one table, and one data file and can be found with this article online at http://dx.doi.org/10.1016/j.cels.2017.06.002.

AUTHOR CONTRIBUTIONS

S.J.B. and C.J.R. wrote code for the database. J.C. contributed R code for statistical analysis. C.J.L. provided guidance on the design of the database and the manuscript. C.J.R. conceived and designed the resource and wrote the manuscript. All authors read and approved the final manuscript.

ACKNOWLEDGMENTS

We thank Lars Juhl Jensen for help with STRING integration. We also thank members of the ICR Gene Function Team and Systems Biology Ireland for providing feedback on earlier versions of this resource. We acknowledge NHS funding to the ICR/Royal Marsden Hospital Biomedical Research Center. Work on this project was supported by a grant to C.J.R. from the Irish Health Research Board (KEDS-2015-1636). C.J.L. is supported by Cancer Research UK (grant number C347/A8363) and Breast Cancer Now. C.J.R. is a Sir Henry Wellcome Fellow jointly funded by Science Foundation Ireland, the Health Research Board, and the Wellcome Trust (grant number 103049/Z/13/Z) under the SFI-HRB-Wellcome Trust Biomedical Research Partnership.

Received: November 29, 2016
Revised: April 4, 2017
Accepted: June 8, 2017
Published: July 12, 2017

REFERENCES

Ashworth, A., Lord, C.J., and Reis-Filho, J.S. (2011). Genetic interactions in cancer progression and treatment. Cell *145*, 30–38.

Barretina, J., Caponigro, G., Stransky, N., Venkatesan, K., Margolin, A.A., Kim, S., Wilson, C.J., Lehar, J., Kryukov, G.V., Sonkin, D., et al. (2012). The Cancer Cell Line Encyclopedia enables predictive modelling of anticancer drug sensitivity. Nature *483*, 603–607.

Basu, A., Bodycombe, N.E., Cheah, J.H., Price, E.V., Liu, K., Schaefer, G.I., Ebright, R.Y., Stewart, M.L., Ito, D., Wang, S., et al. (2013). An interactive resource to identify cancer genetic and lineage dependencies targeted by small molecules. Cell *154*, 1151–1161.

Brough, R., Frankum, J.R., Costa-Cabral, S., Lord, C.J., and Ashworth, A. (2011a). Searching for synthetic lethality in cancer. Curr. Opin. Genet. Dev. *21*, 34–41.

Brough, R., Frankum, J.R., Sims, D., Mackay, A., Mendes-Pereira, A.M., Bajrami, I., Costa-Cabral, S., Rafiq, R., Ahmad, A.S., Cerone, M.A., et al. (2011b). Functional viability profiles of breast cancer. Cancer Discov. *1*, 260–273.

Campbell, J., Ryan, C.J., Brough, R., Bajrami, I., Pemberton, H.N., Chong, I.Y., Costa-Cabral, S., Frankum, J., Gulati, A., Holme, H., et al. (2016). Large-scale profiling of kinase dependencies in cancer cell lines. Cell Rep. *14*, 2490–2501.

Cheung, H.W., Cowley, G.S., Weir, B.A., Boehm, J.S., Rusin, S., Scott, J.A., East, A., Ali, L.D., Lizotte, P.H., Wong, T.C., et al. (2011). Systematic investigation of genetic vulnerabilities across cancer cell lines reveals lineage-specific dependencies in ovarian cancer. Proc. Natl. Acad. Sci. USA *108*, 12372–12377.

Cowley, G.S., Weir, B.A., Vazquez, F., Tamayo, P., Scott, J.A., Rusin, S., East-Seletsky, A., Ali, L.D., Gerath, W.F., Pantel, S.E., et al. (2014). Parallel genome-scale loss of function screens in 216 cancer cell lines for the identification of context-specific genetic dependencies. Sci. Data *1*, 140035.

Daemen, A., Griffith, O.L., Heiser, L.M., Wang, N.J., Enache, O.M., Sanborn, Z., Pepin, F., Durinck, S., Korkola, J.E., Griffith, M., et al. (2013). Modeling precision treatment of breast cancer. Genome Biol. *14*, R110.

Downward, J. (2015). RAS synthetic lethal screens revisited: still seeking the elusive prize? Clin. Cancer Res. *21*, 1802–1809.

Forbes, S.A., Beare, D., Gunasekaran, P., Leung, K., Bindal, N., Boutselakis, H., Ding, M., Bamford, S., Cole, C., Ward, S., et al. (2015). COSMIC: exploring the world's knowledge of somatic mutations in human cancer. Nucleic Acids Res. *43*, D805–D811.

Garnett, M.J., Edelman, E.J., Heidorn, S.J., Greenman, C.D., Dastur, A., Lau, K.W., Greninger, P., Thompson, I.R., Luo, X., Soares, J., et al. (2012). Systematic identification of genomic markers of drug sensitivity in cancer cells. Nature *483*, 570–575.

Gray, K.A., Yates, B., Seal, R.L., Wright, M.W., and Bruford, E.A. (2015). Genenames.org: the HGNC resources in 2015. Nucleic Acids Res. *43*, D1079–D1085.

Griffith, M., Griffith, O.L., Coffman, A.C., Weible, J.V., McMichael, J.F., Spies, N.C., Koval, J., Das, I., Callaway, M.B., Eldred, J.M., et al. (2013). DGIdb: mining the druggable genome. Nat. Methods *10*, 1209–1210.

Helming, K.C., Wang, X., Wilson, B.G., Vazquez, F., Haswell, J.R., Manchester, H.E., Kim, Y., Kryukov, G.V., Ghandi, M., Aguirre, A.J., et al. (2014). ARID1B is a specific vulnerability in ARID1A-mutant cancers. Nat. Med. *20*, 251–254.

Hsu, T.Y., Simon, L.M., Neill, N.J., Marcotte, R., Sayad, A., Bland, C.S., Echeverria, G.V., Sun, T., Kurley, S.J., Tyagi, S., et al. (2015). The spliceosome is a therapeutic vulnerability in MYC-driven cancer. Nature *525*, 384–388.

Hynes, N.E., and Lane, H.A. (2005). ERBB receptors and cancer: the complexity of targeted inhibitors. Nat. Rev. Cancer *5*, 341–354.

Iorio, F., Knijnenburg, T.A., Vis, D.J., Bignell, G.R., Menden, M.P., Schubert, M., Aben, N., Goncalves, E., Barthorpe, S., Lightfoot, H., et al. (2016). A landscape of pharmacogenomic interactions in cancer. Cell *166*, 740–754.

Jackson, A.L., and Linsley, P.S. (2010). Recognizing and avoiding siRNA off-target effects for target identification and therapeutic application. Nat. Rev. Drug Discov. *9*, 57–67.

Kaelin, W.G., Jr. (2012). Molecular biology. Use and abuse of RNAi to study mammalian gene function. Science *337*, 421–422.

Kelley, R., and Ideker, T. (2005). Systematic interpretation of genetic interactions using protein networks. Nat. Biotechnol. *23*, 561–566.

Lord, C.J., Tutt, A.N., and Ashworth, A. (2015). Synthetic lethality and cancer therapy: lessons learned from the development of PARP inhibitors. Annu. Rev. Med. *66*, 455–470.

Luo, J., Solimini, N.L., and Elledge, S.J. (2009). Principles of cancer therapy: oncogene and non-oncogene addiction. Cell *136*, 823–837.

Marcotte, R., Brown, K.R., Suarez, F., Sayad, A., Karamboulas, K., Krzyzanowski, P.M., Sircoulomb, F., Medrano, M., Fedyshyn, Y., Koh, J.L., et al. (2012). Essential gene profiles in breast, pancreatic, and ovarian cancer cells. Cancer Discov. *2*, 172–189.

Marcotte, R., Sayad, A., Brown, K.R., Sanchez-Garcia, F., Reimand, J., Haider, M., Virtanen, C., Bradner, J.E., Bader, G.D., Mills, G.B., et al. (2016). Functional genomic landscape of human breast cancer drivers, vulnerabilities, and resistance. Cell *164*, 293–309.

Shao, D.D., Tsherniak, A., Gopal, S., Weir, B.A., Tamayo, P., Stransky, N., Schumacher, S.E., Zack, T.I., Beroukhim, R., Garraway, L.A., et al. (2013). ATARiS: computational quantification of gene suppression phenotypes from multisample RNAi screens. Genome Res. *23*, 665–678.

Szklarczyk, D., Franceschini, A., Wyder, S., Forslund, K., Heller, D., Huerta-Cepas, J., Simonovic, M., Roth, A., Santos, A., Tsafou, K.P., et al. (2015). STRING v10: protein-protein interaction networks, integrated over the tree of life. Nucleic Acids Res. *43*, D447–D452.

Vogelstein, B., Papadopoulos, N., Velculescu, V.E., Zhou, S., Diaz, L.A., Jr., and Kinzler, K.W. (2013). Cancer genome landscapes. Science *339*, 1546–1558.

Wagner, A.H., Coffman, A.C., Ainscough, B.J., Spies, N.C., Skidmore, Z.L., Campbell, K.M., Krysiak, K., Pan, D., McMichael, J.F., Eldred, J.M., et al. (2016). DGIdb 2.0: mining clinically relevant drug-gene interactions. Nucleic Acids Res. *44*, D1036–D1044.

Wang, T., Yu, H., Hughes, N.W., Liu, B., Kendirli, A., Klein, K., Chen, W.W., Lander, E.S., and Sabatini, D.M. (2017). Gene essentiality profiling reveals gene networks and synthetic lethal interactions with oncogenic Ras. Cell *168*, 890–903.e815.

Yang, W., Soares, J., Greninger, P., Edelman, E.J., Lightfoot, H., Forbes, S., Bindal, N., Beare, D., Smith, J.A., Thompson, I.R., et al. (2013). Genomics of Drug Sensitivity in Cancer (GDSC): a resource for therapeutic biomarker discovery in cancer cells. Nucleic Acids Res. *41*, D955–D961.

STAR★METHODS

KEY RESOURCES TABLE

REAGENT or RESOURCE	SOURCE	IDENTIFIER
Deposited Data		
Cell Line Copy Number Data	Iorio et al., 2016	ftp://ftp.sanger.ac.uk/pub/project/cancerrxgene/releases/release-6.0/Gene_level_CN.xlsx
Cell Line Exome Data	Iorio et al., 2016	http://www.cancerrxgene.org/gdsc1000/GDSC1000_WebResources//Data/suppData/TableS2C.xlsx
Achilles v2 shRNA data	Cowley et al., 2014	https://ndownloader.figshare.com/files/3178886
COLT shRNA data	Marcotte et al., 2012	http://dpsc.ccbr.utoronto.ca/cancer/datasets.html; RRID: SCR_006485
Wang et al., CRISPR data	Wang et al., 2017	Table S3 in Wang et al.
Marcotte et al., 2016 Breast Cancer shRNA data	Marcotte et al., 2016	https://github.com/neellab/bfg/blob/gh-pages/data/shrna/breast_zgarp.txt.zip?raw=true
Kinome siRNA data	Campbell et al., 2016	Table S1B
STRING interactions	Szklarczyk et al., 2015	http://string-db.org/download/protein.links.v10/9606.protein.links.v10.txt.gz; RRID: SCR_005223
Drug Target Information	Wagner et al., 2016	http://dgidb.genome.wustl.edu/; RRID: SCR_006608
Gene names and synonyms	Gray et al., 2015	ftp://ftp.ebi.ac.uk/pub/databases/genenames/new/tsv/hgnc_complete_set.txt; RRID: SCR_002827
Software and Algorithms		
Django	Django Software Foundation	https://www.djangoproject.com/; RRID: SCR_012855
JQuery	jQuery Foundation	https://jquery.com/
Python version 3.4	Python Software Foundation	https://www.python.org/; RRID: SCR_008394
R version 3.3.1	R Foundation for Statistical Computing	https://www.r-project.org/; RRID: SCR_001905
Intercell Analysis Scripts	Campbell et al., 2016	https://github.com/GeneFunctionTeam/kinase-dependency-profiling
CancerGD Python / Javascript / R / HTML code	This study	https://github.com/cancergenetics/cancergd

CONTACT FOR REAGENT AND RESOURCE SHARING

Further information and requests for resources and reagents should be directed to and will be fulfilled by the Lead Contact, Colm J. Ryan (colm.ryan@ucd.ie)

METHOD DETAILS

Genotype Data

Exome data for ~1,000 cell lines are obtained from the GDSC resource (Iorio et al., 2016; Yang et al., 2013). We use this data to annotate ~500 driver genes (Campbell et al., 2016) according to whether they feature likely functional alterations. For oncogenes we consider recurrent missense or recurrent in frame deletions/insertions to be likely functional alterations, where recurrence is defined as at least 3 previous mutations of a particular site in the COSMIC database (Forbes et al., 2015). In addition to recurrent missense or indel events, for tumor suppressors we consider that all nonsense, frameshift and splice-site mutations are likely functional alterations. For copy number analysis we use the gene level copy number scores from COSMIC for the same set of cell lines (which are derived from PICNIC analysis of Affymetrix SNP6.0 array data) (Forbes et al., 2015; Garnett et al., 2012; Iorio et al., 2016; Yang et al., 2013). An oncogene is considered amplified if the entire coding sequence has 8 or more copies while a tumor suppressor is considered deleted if any part of the coding sequence has a copy number of 0 as per Garnett et al (Garnett et al., 2012). For the majority of driver genes we integrate the two sources together. For all tumor suppressors we consider a functional alteration to be either a deletion (derived from copy number profiles) or a presumed loss-of-function mutation (as identified in the exome data). For most oncogenes we consider a functional alteration to be either an amplification or a recurrent mutation/indel. For a small number of oncogenes (*ERBB2, MYC, MYCN*) we consider only amplifications as functional events, while for another group (*KRAS, BRAF, NRAS, HRAS*) we only consider recurrent mutations/indels.

Loss of Function Screens

Four large-scale RNAi datasets and one CRISPR dataset are currently included in CancerGD (Campbell et al., 2016; Cowley et al., 2014; Marcotte et al., 2012, 2016; Wang et al., 2017). These include a kinome focussed siRNA screen covering a panel of 117 cell lines from diverse histologies (Campbell et al., 2016), a genome-scale shRNA screen focussed on 77 breast tumor cell lines (Marcotte et al., 2016), a genome-scale shRNA screen focussed on 72 breast, ovarian and pancreatic cell lines(Marcotte et al., 2012), a large-scale shRNA screen covering 216 cell lines from diverse histologies (Cowley et al., 2014), and a genome-scale CRISPR screen covering 14 AML cell lines(Wang et al., 2017). Cowley et al (Cowley et al., 2014) is largely a superset of a previous screen from the same lab (Cheung et al., 2011) and hence the two resources are not included separately. Similarly the kinome siRNA screen from Cambell et al (Campbell et al., 2016) contains the majority of the breast tumor cell lines screened in a previous breast cancer kinome siRNA screen from the same lab (Brough et al., 2011b) and hence they are not included separately. The breast cell lines in (Marcotte et al., 2016) are a superset of those included in (Marcotte et al., 2012) and consequently we do not store breast specific dependencies from (Marcotte et al., 2012).

Cell Line Naming

Internally we follow the naming convention established by the Cancer Cell Line Encyclopedia (Barretina et al., 2012). The CCLE naming convention is the cell line name (containing only numbers and upper case letters) followed by an underscore, followed by the tissue/primary site in upper case. The cell line names are taken from (Iorio et al., 2016), converted to uppercase and punctuation removed. Where possible we use the same tissue types as the CCLE, in a small number of cases where a tissue was absent from the CCLE (e.g. CERVIX) we have created a new tissue type. Having the tissue type in the cell line name facilitates filtering the boxplots (e.g. to show the gene inhibition sensitivities for cell lines from a specific tissue) in the browser without having to perform additional database queries. Furthermore two of the published loss-of-function screens already follow this naming convention (Campbell et al., 2016; Cowley et al., 2014) while a third features only breast cell lines and was trivially converted (Marcotte et al., 2016). In instances where the same cell line is featured in two datasets but there is a naming disagreement (e.g. H1299_LUNG in Campbell et al (Campbell et al., 2016) is NCIH1299_LUNG in our genotype set) we manually rename the screen dataset to match the genotype data.

Gene Identification

CancerGD provides links to multiple external sources that use a variety of different gene identifiers. Consequently for each gene in the database we store multiple identifiers (Entrez Gene ID, Ensembl Gene identifiers, HUGO Gene Names, Ensembl Protein IDs). We also store synonyms for each gene to facilitate easy gene look up (e.g. *ERBB2* can be identified by searching for *HER2*). These synonyms are obtained from the HGNC resource (Gray et al., 2015).

Drug Target Annotations

Drug-gene relationships are obtained from the Drug-Gene Interaction Database (DGIdb), which integrates drug-gene relationships from multiple sources (Wagner et al., 2016). Only inhibitor relationships are retrieved, as we are interested in drugs that inhibit the products of specific genes, rather than drugs whose efficacy is associated with the mutation of specific genes. Results from DGIdb sourced from MyCancerGenome and MyCancerGenomeClinicalTrial are excluded for the same reason.

Functional Interactions

Functional interactions are obtained from STRING. We store all interactions that are medium confidence (STRING score > 0.4) or higher. Cut-offs to identify interactions as ‘Medium’, ‘High’ and ‘Highest’ confidence are those defined by STRING. For displaying the functional interactions between the dependencies associated with each driver gene we use the STRING API (Szklarczyk et al., 2015).

Implementation

CancerGD is implemented in Python using the Django framework and follows a model/view/controller architecture. JQuery is used for Javascript processing in the browser interface. MySQL is used by default for data storage but SQLite can be used for development / testing purposes with minimal documented changes. The application is currently hosted on the PythonAnywhere system, a generic Python web services host, suggesting that the application is portable.

Formatting Screens for CancerGD

To enable easy inclusion of future screens in CancerGD we request that data be provided as a tab-delimited table with each row representing a particular cell line and each column representing reagents targeting a specific gene. Cell line names should preferably follow the Cancer Cell Line Encyclopaedia naming convention described above, but COSMIC IDs are also acceptable. Genes should preferably be identified using ENTREZ IDs but other unique IDs (ENSEMBL Gene IDs) are acceptable. Due to regular changing and updating, gene symbols alone should not be used as unique gene identifiers. We favour SYMBOL_ENTREZID (e.g KRAS_3846) for ease of use but this is not required. In cases where multiple distinct scores are provided for a specific gene, as happens with scores from the ATARIS algorithm, we request that they be identified using distinct suffixes (e.g. KRAS_3846_1, KRAS_3846_2).

Individual entries in the table should be quantitative scores indicating how sensitive a specific cell line is to perturbation of a particular gene. As different scoring procedures are used to quantify the results of screens using different experimental approaches

(e.g. ATARIS (Shao et al., 2013) and zGARP (Marcotte et al., 2012) for shRNA screens, Z score for siRNA screens (Campbell et al., 2016)) we do not require the scores to be in any standard format or range. However, we follow the convention in the field and suggest that increasingly negative scores should indicate greater inhibition of cell growth. A sample screen from Campbell et al (Campbell et al., 2016) is provided in the appropriate format here: http://www.cancergd.org/static/gendep/Campbell_cancergd.txt

QUANTIFICATION AND STATISTICAL ANALYSIS

We use R for all statistical analysis. For each driver gene / target gene combination we compare cell lines harbouring a likely functional alteration in the driver gene to cell lines with no alteration in that gene and test if the cell lines with the functional alteration are more sensitive to RNAi reagents that inhibit that gene. This is tested using a one-sided Mann-Whitney U test. A variety of alternative two-sample tests have been used in previous publications, including median permutation tests (Brough et al., 2011b; Campbell et al., 2016) and mutual information based measures (Cowley et al., 2014). The Mann-Whitney U test has a number of advantages for CancerGD – it is rapid to calculate and it does not assume that the scores for each gene are normally distributed. The latter is important as it means the test can be used uniformly on loss-of-function screens from multiple sources that use different scoring schemes. For all screens we use the authors' provided scoring scheme (zGARP for Marcotte et al (Marcotte et al., 2012; Marcotte et al., 2016), ATARIS phenotype score for Cowley et al (Cowley et al., 2014), robust Z score for Campbell et al (Campbell et al., 2016), and CS score for Wang et al (Wang et al., 2017)). As in (Marcotte et al., 2012) we apply Z score normalization to the zGARP scores from (Marcotte et al., 2012) to enable reasonable comparison of scores across cell lines. In addition to the p-value from the Mann-Whitney U test we calculate a common language effect size (CLES) for each dependency. The CLES is equivalent to the *Area under the ROC curve* and the *Probability of Superiority* and indicates the probability that a cell line with an alteration in a particular driver gene is more sensitive to a given RNAi reagent than a cell line without that alteration. In the database we store all nominally significant dependencies ($p<0.05$) with a CLES ≥ 0.65. In a small number of instances multiple ATARIS scores are presented for a single gene – when storing CGDs we incorporate the ATARIS score with the lower p-value.

DATA AND SOFTWARE AVAILABILITY

Source code for the entire project (R/Python/Javascript/HTML) is publicly available on GitHub (https://github.com/cancergenetics/cancergd). Detailed instructions on how to run the statistical analysis, install the web application and populate the database are also provided in the GitHub repository.

Cell Systems, Volume 5

Supplemental Information

CancerGD: A Resource for Identifying and Interpreting Genetic Dependencies in Cancer

Stephen Bridgett, James Campbell, Christopher J. Lord, and Colm J. Ryan

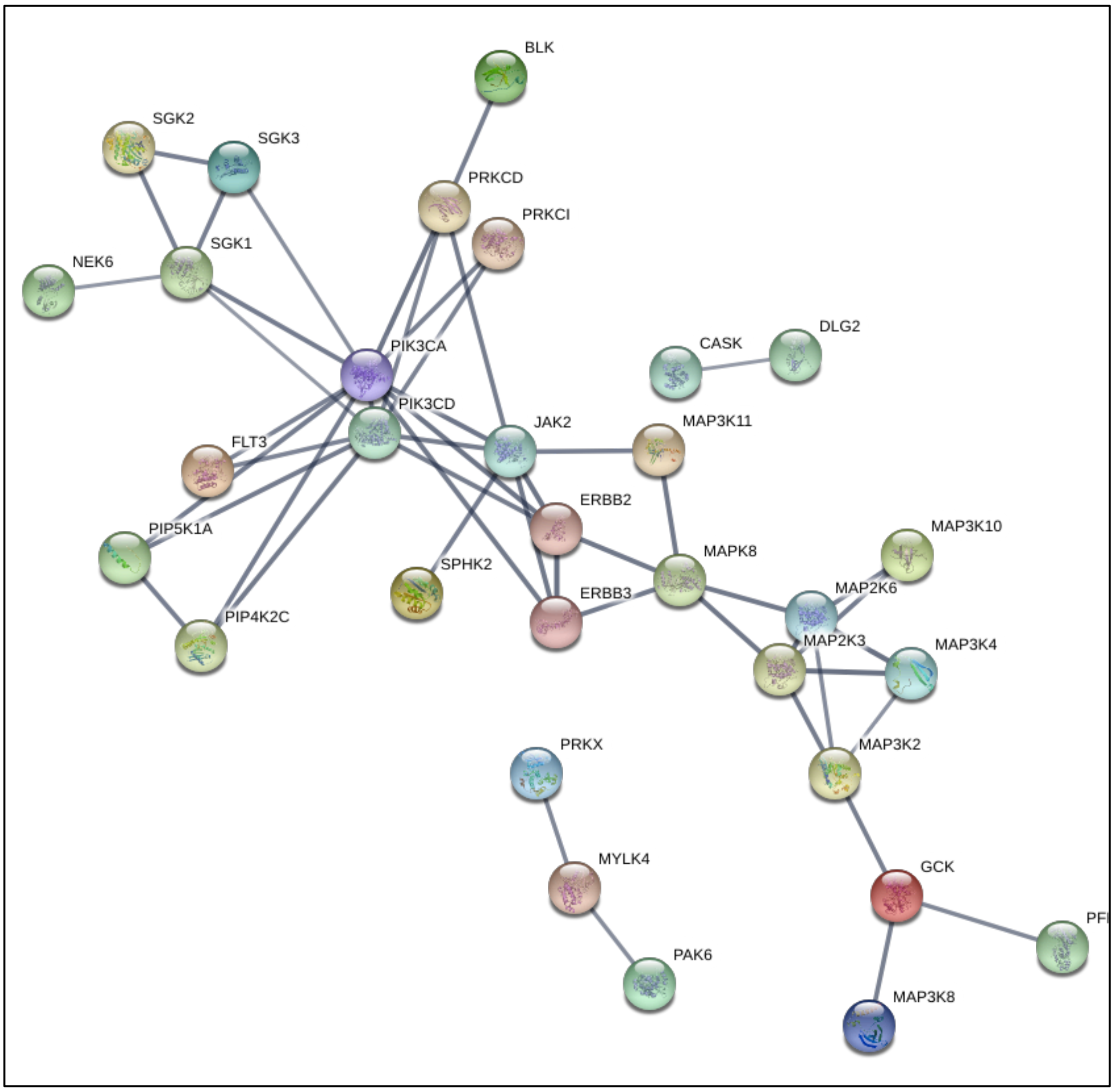

Figure S1. Visualizing the known interactions between all CGDs associated with a specific driver gene. Related to Figure 2.

High confidence STRING functional interactions between CGDs associated with *ERBB2* amplification in Campbell *et al* are shown.

Table S1. Driver genes currently included in CancerGD. Related to Figure 1

Gene	Studies	Tissues	CGDS
ACVR2A	1	2	676
AFDN	1	1	396
AFF4	1	1	268
APC	1	2	905
ARID1A	4	5	2336
ARID1B	2	2	273
ASXL1	2	2	1648
B2M	1	1	294
BCOR	1	2	389
BRAF	1	2	562
BRCA1	1	1	798
BRCA2	2	3	1685
CCND1	3	3	1264
CCNE1	1	1	930
CDH1	3	2	1761
CDKN2A	5	8	4074
CDKN2C	1	3	1148
CTNNB1	1	2	455
EGFR	2	2	460
EP300	2	3	1698
ERBB2	4	3	2523
EZH2	1	1	587
FANCA	1	2	707
FBXW7	1	2	440
GNAS	2	3	1434
HEY1	1	1	643
KDM6A	1	3	743
KRAS	3	4	2205
MAP2K4	2	2	1048
MDM2	1	1	224
MSH2	1	1	172
MSH6	1	2	651
MYC	3	5	1782
NCOA3	1	1	931
NCOR1	1	2	540
NF1	3	4	1271
NRAS	2	2	1232
PIK3CA	4	5	2800
PIK3R1	1	2	372
PPM1D	3	1	1156
PTCH1	1	1	197

PTEN	4	4	2176
PTPRK	1	1	289
RB1	3	3	1602
RNF43	2	3	1787
RPL22	1	2	836
SKP2	1	1	1095
SMAD4	3	4	1863
SMARCA4	2	2	437
SPOP	1	1	738
STK11	2	2	341
TP53	5	9	3690
UBR5	1	2	677

Methods S1. CancerGD short tutorial (20 mins)

Overview:

CancerGD.org provides a search interface for genetic dependencies identified in loss-of-function screens in panels of tumor cell lines. A genetic dependency is identified when there is a statistical association between the presence of a particular mutation and increased sensitivity to the inhibition of a specific gene. These dependencies are identified by integrating large-scale loss-of-function screens in panels of cell lines with genotype data for the same cell lines. In CancerGD we store all nominally significant dependencies ($P < 0.05$) with a common language effect size > 65% (see http://www.cancergd.org/faq/#effectsize for an explanation). A goal of this resource is to help understand genetic dependencies in the context of known functional interaction networks (e.g. protein-protein interactions). Towards this end we have developed simple functionality to identify those genetic dependencies that occur **within pathways** (i.e. where the driver gene and the target dependency belong to the same pathway) and **between pathways** (i.e. where the dependencies associated with a given driver gene belong to the same complex or pathway as each other). To further facilitate follow on studies we have also annotated all dependencies in the database according to the availability of inhibitors for the target genes.

Here we provide a simple tutorial that takes the user through the main functionality of www.cancergd.org. We show how CancerGD can be used to browse and analyse the dependencies associated with *ERBB2* amplification in the Campbell *et al* paper published in Cell Reports (2016). This tutorial should take approximately 20 minutes to complete.

Step 1 – retrieving the dependencies associated with a driver gene

Navigate to http://www.cancergd.org/ in your internet browser. You will see a search box resembling the below image. In the **Driver gene** field type *'ERBB2'*, in the **Tissue type** dropdown select *'Pan cancer'* and in the **Study** dropdown please select *'Campbell(2016)'*. Click the **Search** button

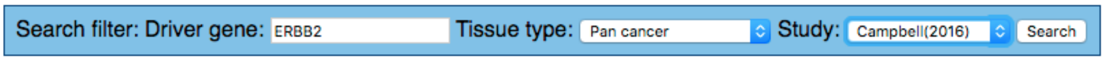

You will be presented with a table of results resembling the below image. The top of the page provides details (gene synonyms, a gene description, links to the gene on external resources) for the selected driver gene (*ERBB2)*. The bottom of the page is a table displaying all of the nominally significant dependencies associated with the selected driver gene (*ERBB2*) in the selected tissue (pan-cancer, i.e. across all tissue types) from the selected study (Campbell *et al*).

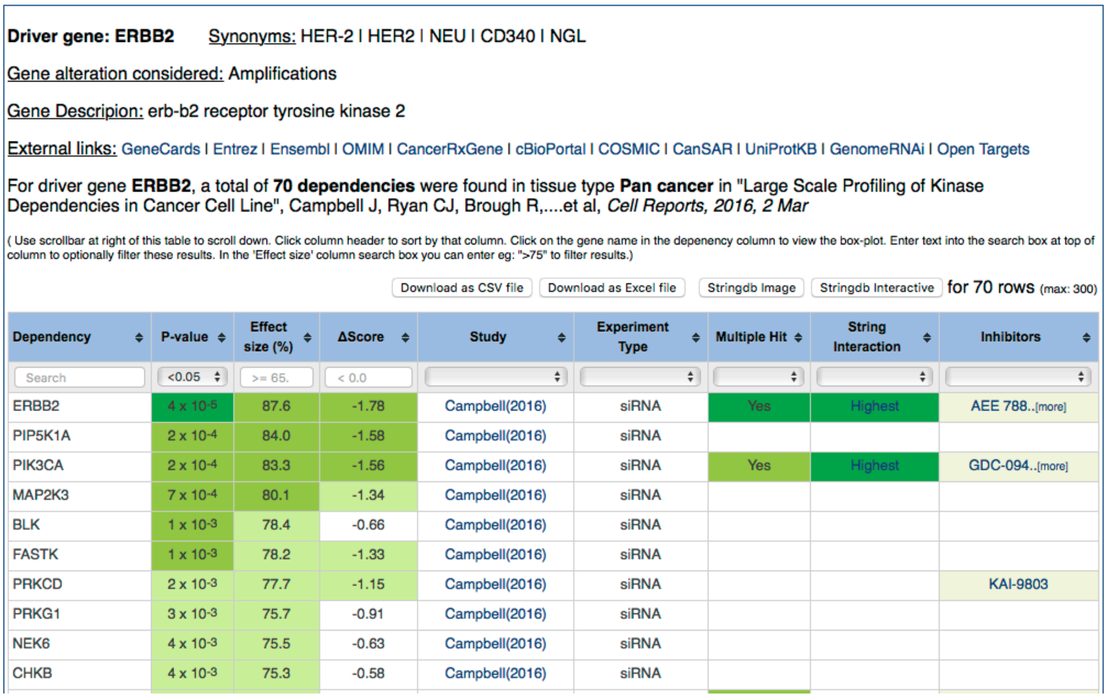

Driver gene: ERBB2 Synonyms: HER-2 I HER2 I NEU I CD340 I NGL

Gene alteration considered: Amplifications

Gene Descripion: erb-b2 receptor tyrosine kinase 2

External links: GeneCards I Entrez I Ensembl I OMIM I CancerRxGene I cBioPortal I COSMIC I CanSAR I UniProtKB I GenomeRNAi I Open Targets

For driver gene **ERBB2**, a total of **70 dependencies** were found in tissue type **Pan cancer** in "Large Scale Profiling of Kinase Dependencies in Cancer Cell Line", Campbell J, Ryan CJ, Brough R,....et al, *Cell Reports, 2016, 2 Mar*

(Use scrollbar at right of this table to scroll down. Click column header to sort by that column. Click on the gene name in the depenency column to view the box-plot. Enter text into the search box at top of column to optionally filter these results. In the 'Effect size' column search box you can enter eg: ">75" to filter results.)

Download as CSV file | Download as Excel file | Stringdb Image | Stringdb Interactive for 70 rows (max: 300)

Dependency	P-value	Effect size (%)	ΔScore	Study	Experiment Type	Multiple Hit	String Interaction	Inhibitors
Search	<0.05	>= 65.	< 0.0					
ERBB2	4×10^{-5}	87.6	-1.78	Campbell(2016)	siRNA	Yes	Highest	AEE 788..[more]
PIP5K1A	2×10^{-4}	84.0	-1.58	Campbell(2016)	siRNA			
PIK3CA	2×10^{-4}	83.3	-1.56	Campbell(2016)	siRNA	Yes	Highest	GDC-094..[more]
MAP2K3	7×10^{-4}	80.1	-1.34	Campbell(2016)	siRNA			
BLK	1×10^{-3}	78.4	-0.66	Campbell(2016)	siRNA			
FASTK	1×10^{-3}	78.2	-1.33	Campbell(2016)	siRNA			
PRKCD	2×10^{-3}	77.7	-1.15	Campbell(2016)	siRNA			KAI-9803
PRKG1	3×10^{-3}	75.7	-0.91	Campbell(2016)	siRNA			
NEK6	4×10^{-3}	75.5	-0.63	Campbell(2016)	siRNA			
CHKB	4×10^{-3}	75.3	-0.58	Campbell(2016)	siRNA			

Clicking on any gene name in the 'Dependency' column will present the user with a view of the evidence supporting that dependency. Click on *MAP2K3* to proceed to the next step

Step 2 – viewing the data supporting individual dependencies

You will be presented with a window resembling the below image. This view presents the data supporting the association between *ERBB2* amplification and sensitivity to RNAi reagents targeting *MAP2K3*.

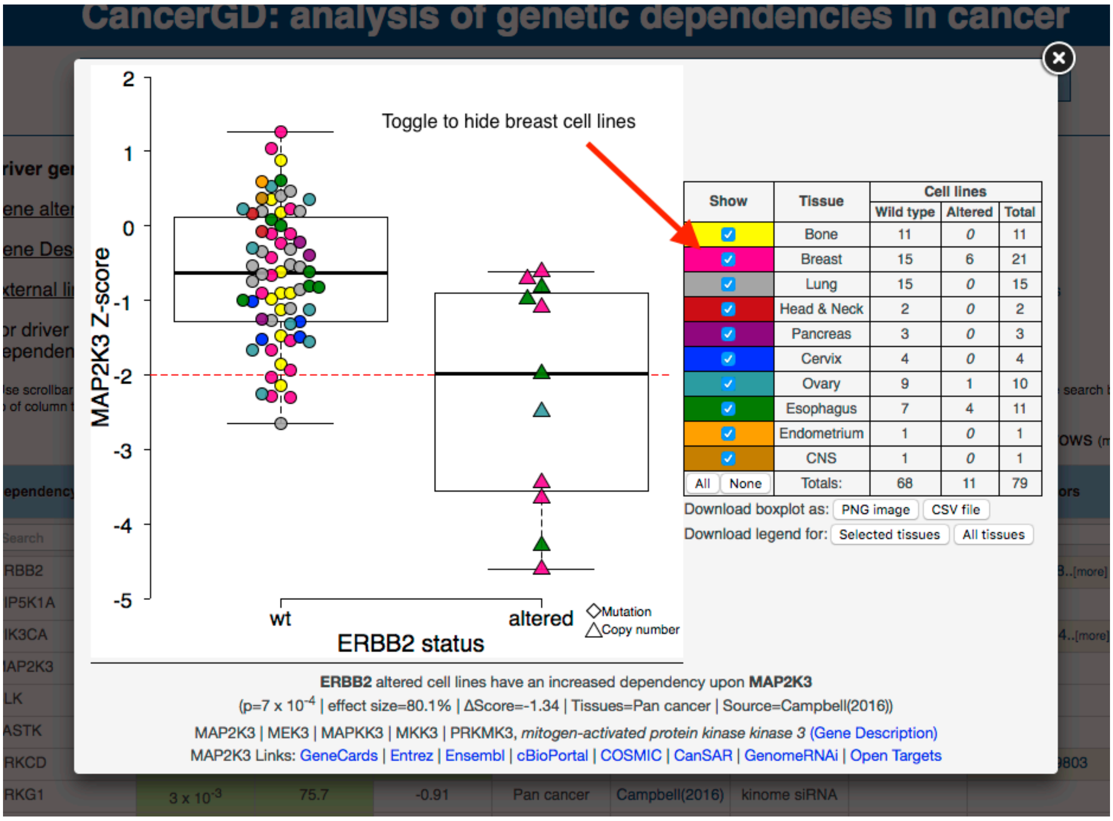

This is an interactive box plot (http://www.cancergd.org/faq/#boxplots) that displays the sensitivity of cell lines partitioned according to *ERBB2* status to RNAi reagents targeting *MAP2K3*. The cell lines featuring an alteration of *ERBB2* are displayed on the right and the cell lines without the alteration are on the left. Each colored shape represents a cell line and the position along the y-axis indicates how sensitive that cell line is to the RNAi reagents targeting the gene indicated (*MAP2K3)*. A lower position on the y-axis indicates greater sensitivity. The colors indicate the tissue of origin for each cell line, as indicated in the legend on the right hand side. Toggles in the legend facilitate hiding or displaying cell lines from specific histologies. To see how the dependency between *ERBB2* and *MAP2K3* appears when breast cell lines are removed uncheck the box beside ‘Breast’ in the legend.

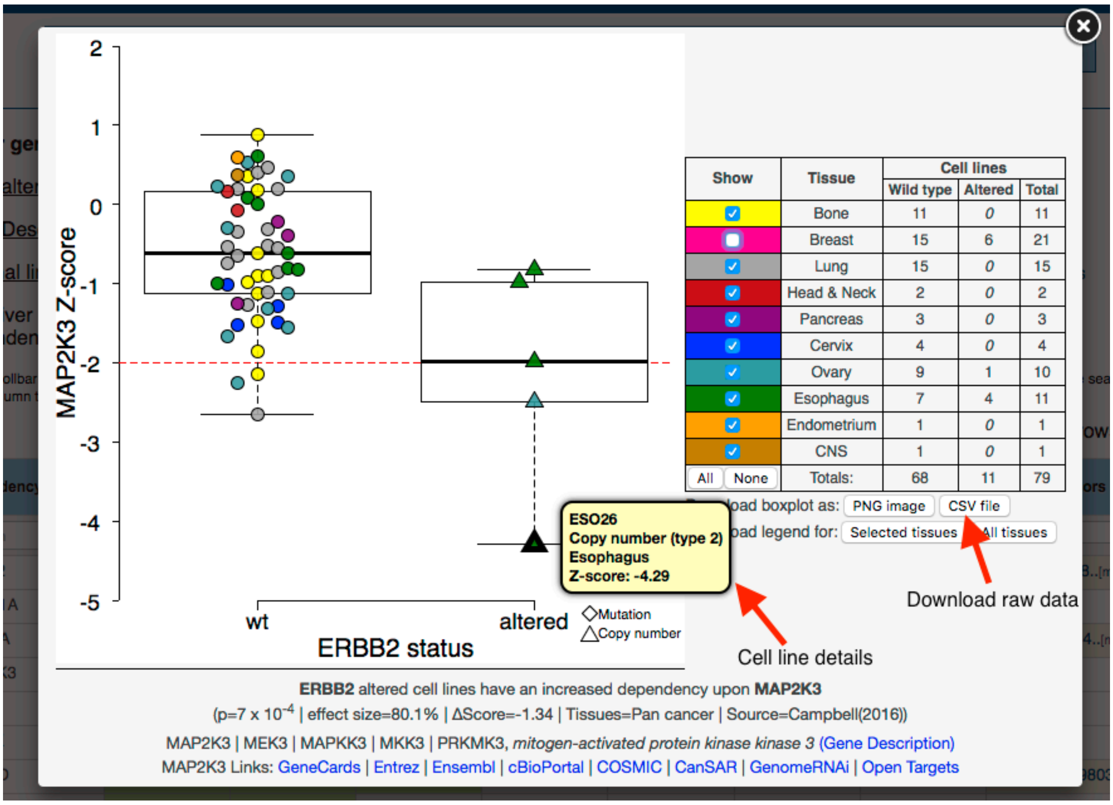

To download a high-resolution copy of this image click 'Download boxplot as **PNG image**'. To download the raw data supporting this dependency in a comma separated text file, click 'Download boxplot as **CSV file'**. This can be opened with Microsoft Excel or similar applications.

To see the details associated with a specific cell line hover your cursor over the shape corresponding to that cell line (e.g. above we hover over the cell line with the greatest sensitivity to *MAP2K3* inhibition).

Click the **X** in the top right to close this image and return to the table that lists genetic dependencies.

Step 3 – filtering dependencies with a known functional relationship to the driver gene

One of the goals of this resource is to facilitate the interpretation of genetic dependencies and to develop filters to prioritize promising candidates for follow up studies. The simplest approach is to focus on dependencies that have a known relationship (e.g. a protein-protein interaction) with the driver gene. To identify these - choose *'Any'* in the *'String Interaction'* column. This will filter the table to show only the genetic dependencies that have a functional relationship (e.g. protein-protein interaction) with *ERBB2* as displayed below.

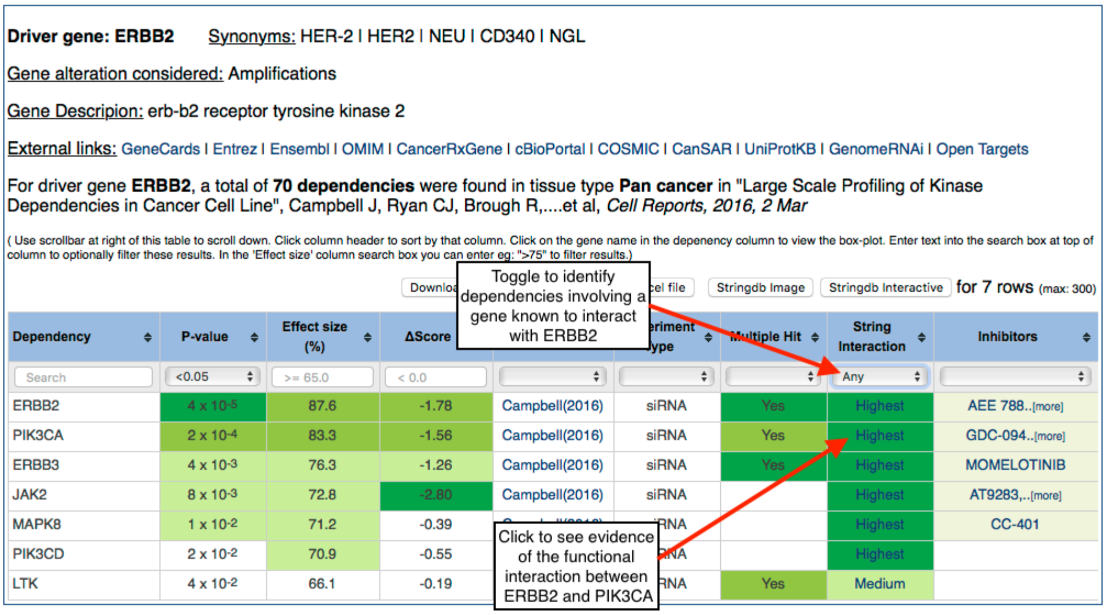

This identifies the *ERBB2* downstream effector *PIK3CA* and the *ERBB2* binding partner *ERBB3* among others. These functional relationships are obtained from the STRING database (http://string-db.org/). Clicking on text inside the *String Interaction* column (e.g. *Highest*) will bring the user to the STRING database where the data supporting the functional interaction between the driver gene and the dependency will be displayed.

Step 4 – identifying interactions between the dependencies associated with a driver gene

An alternative to identifying the known functional interactions between a driver gene and its dependencies is to try to understand the relationship between all of the dependencies associated with a given driver gene. In this way it may be possible to identify pathways or protein complexes upon which the driver gene is associated with an increased dependency. For this analysis we again rely on the STRING database (http://string-db.org/). To view all of the interactions between the dependencies associated *ERBB2* click on the **'Stringdb Image'** button above the dependencies table.

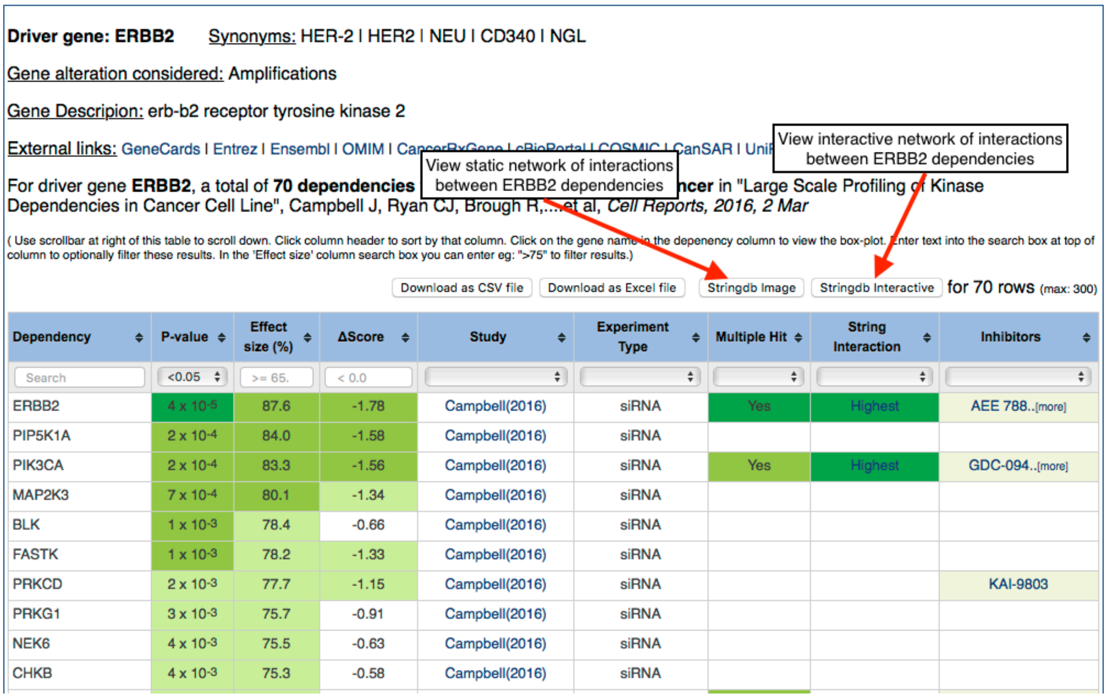

Driver gene: ERBB2 Synonyms: HER-2 | HER2 | NEU | CD340 | NGL

Gene alteration considered: Amplifications

Gene Descripion: erb-b2 receptor tyrosine kinase 2

External links: GeneCards | Entrez | Ensembl | OMIM | [illegible] | CanSAR | [illegible]

For driver gene **ERBB2**, a total of **70 dependencies** [illegible] in "Large Scale Profiling of Kinase Dependencies in Cancer Cell Line", Campbell J, Ryan CJ, Brough R,....et al, *Cell Reports, 2016, 2 Mar*

(Use scrollbar at right of this table to scroll down. Click column header to sort by that column. Click on the gene name in the depenency column to view the box-plot. Enter text into the search box at top of column to optionally filter these results. In the 'Effect size' column search box you can enter eg: ">75" to filter results.)

Download as CSV file | Download as Excel file | Stringdb Image | Stringdb Interactive for 70 rows (max: 300)

Dependency	P-value	Effect size (%)	ΔScore	Study	Experiment Type	Multiple Hit	String Interaction	Inhibitors
Search	<0.05	>= 65.	< 0.0					
ERBB2	4×10^{-5}	87.6	-1.78	Campbell(2016)	siRNA	Yes	Highest	AEE 788..[more]
PIP5K1A	2×10^{-4}	84.0	-1.58	Campbell(2016)	siRNA			
PIK3CA	2×10^{-4}	83.3	-1.56	Campbell(2016)	siRNA	Yes	Highest	GDC-094..[more]
MAP2K3	7×10^{-4}	80.1	-1.34	Campbell(2016)	siRNA			
BLK	1×10^{-3}	78.4	-0.66	Campbell(2016)	siRNA			
FASTK	1×10^{-3}	78.2	-1.33	Campbell(2016)	siRNA			
PRKCD	2×10^{-3}	77.7	-1.15	Campbell(2016)	siRNA			KAI-9803
PRKG1	3×10^{-3}	75.7	-0.91	Campbell(2016)	siRNA			
NEK6	4×10^{-3}	75.5	-0.63	Campbell(2016)	siRNA			
CHKB	4×10^{-3}	75.3	-0.58	Campbell(2016)	siRNA			

This will take a moment to retrieve an image similar to that below showing high-confidence functional interactions between the genes identified as *ERBB2* dependencies. You can see that *ERBB2* amplification is associated with an increased dependency upon a group of kinases functionally related to *ERBB2* and *PI3K* signaling, as well as a group of genes involved in map kinase signaling.

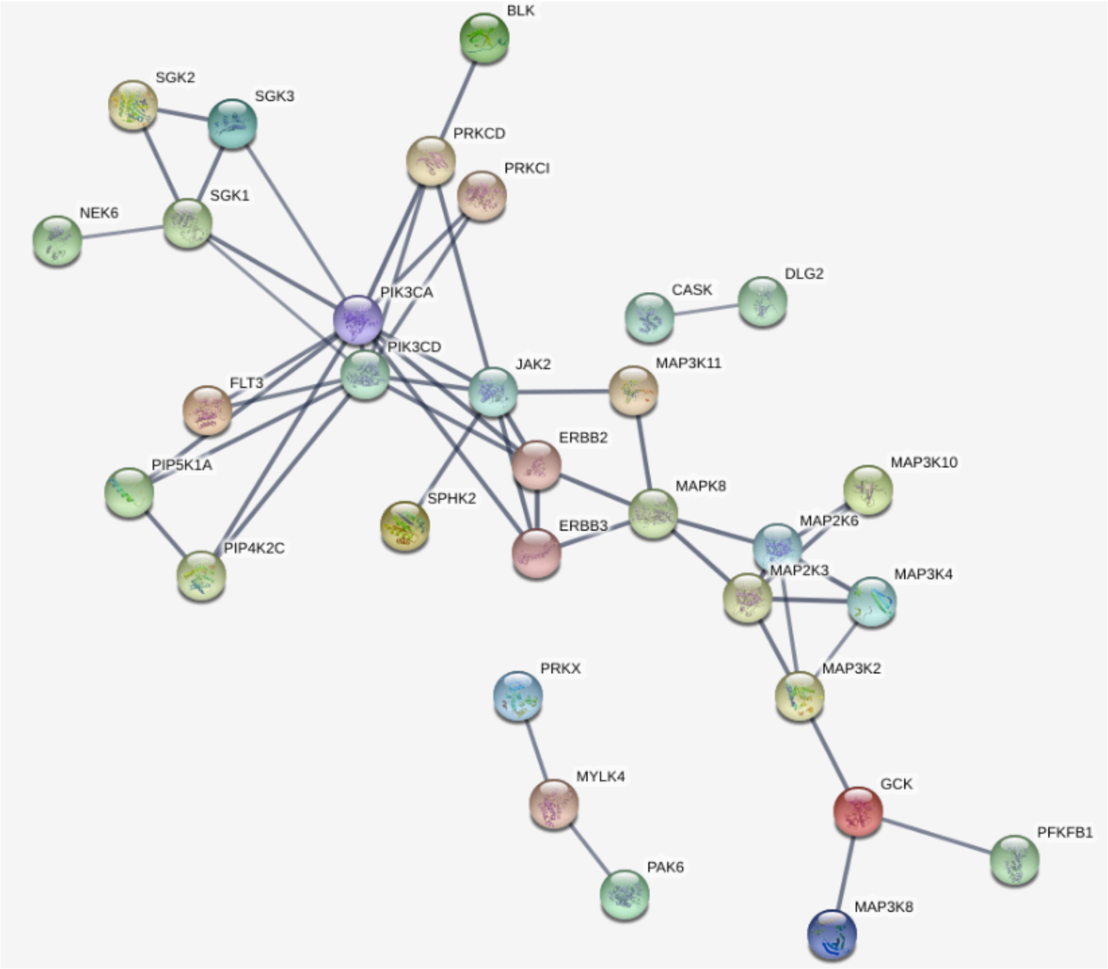

By selecting ‘**Stringdb Interactive**’ instead of **‘Stringdb image’** you can view an interactive version of this network on the STRING website. This will allow you to view the evidence supporting each functional interactions, to alter the layout of the network, and to filter the network in different ways. Click the **X** in the top right of the Stringdb image to close the image and return to the table listing dependencies.

Step 5 – identifying dependencies that can be exploited with existing inhibitors

A further goal of CancerGD is to facilitate follow on experimentation. One means to further explore or validate a dependency is to see if the same effect is observed using small molecule inhibitors rather than RNAi reagents. To that end we annotate all of our dependencies according to the availability of inhibitors. To view genes with available inhibitors, select '*Any'* in the '*Inhibitors'* column toggle. You will see a view resembling the below.

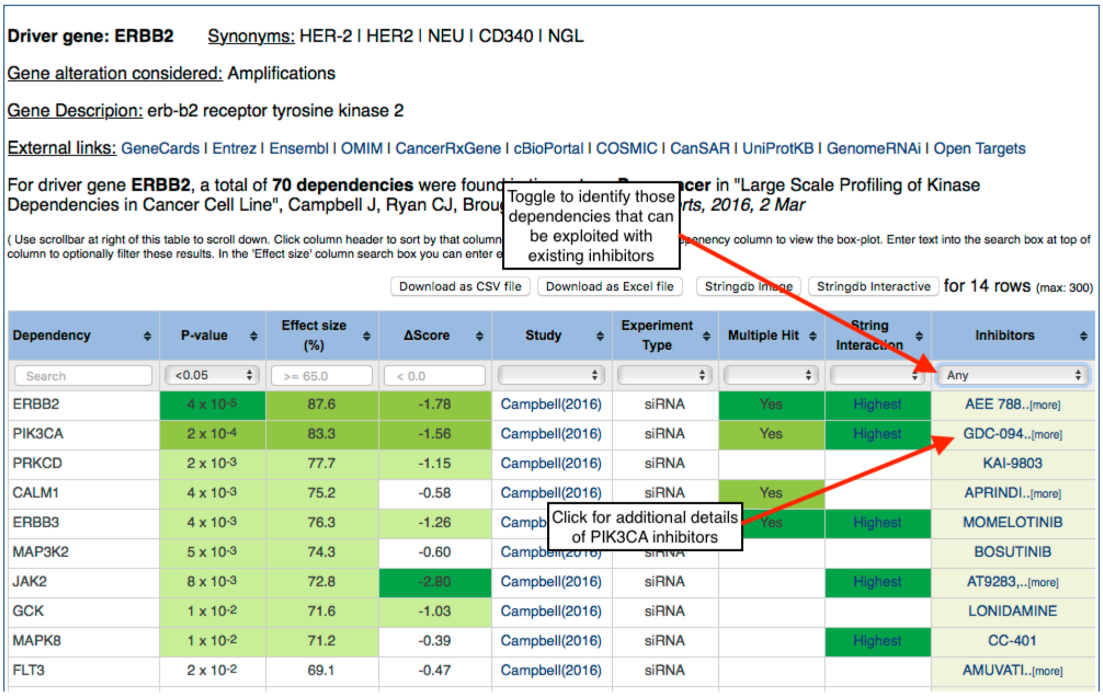

Dependency	P-value	Effect size (%)	ΔScore	Study	Experiment Type	Multiple Hit	String Interaction	Inhibitors
Search	<0.05	>= 65.0	< 0.0					Any
ERBB2	4 x 10-5	87.6	-1.78	Campbell(2016)	siRNA	Yes	Highest	AEE 788..[more]
PIK3CA	2 x 10-4	83.3	-1.56	Campbell(2016)	siRNA	Yes	Highest	GDC-094..[more]
PRKCD	2 x 10-3	77.7	-1.15	Campbell(2016)	siRNA			KAI-9803
CALM1	4 x 10-3	75.2	-0.58	Campbell(2016)	siRNA	Yes		APRINDI..[more]
ERBB3	4 x 10-3	76.3	-1.26	Campb[illegible]	[illegible]	Yes	Highest	MOMELOTINIB
MAP3K2	5 x 10-3	74.3	-0.60	Campb[illegible]	[illegible]			BOSUTINIB
JAK2	8 x 10-3	72.8	-2.80	Campbell(2016)	siRNA		Highest	AT9283,..[more]
GCK	1 x 10-2	71.6	-1.03	Campbell(2016)	siRNA			LONIDAMINE
MAPK8	1 x 10-2	71.2	-0.39	Campbell(2016)	siRNA		Highest	CC-401
FLT3	2 x 10-2	69.1	-0.47	Campbell(2016)	siRNA			AMUVATI..[more]

This filters the dependencies so that only those genes with known inhibitors are presented. The mapping from genes to inhibitors is taken from the DGIdb resource (http://dgidb.genome.wustl.edu/). Clicking on any inhibitor name in the *Inhibitors* column will bring the user to DGIdb, where details on the inhibitor are provided. For some genes there are more inhibitors available than can be presented in the *Inhibitors* column. These are indicated with the text *[more]*. Clicking on *[more]* in any entry in the *Inhibitors* column will display the full list of inhibitors associated with that gene in a window like that shown below :

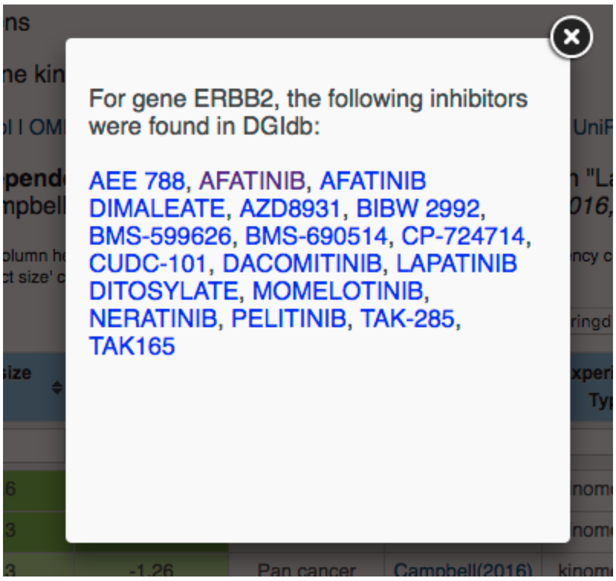

Clicking any inhibitor name within this window will bring the user to DGIdb, where details on the inhibitor are provided. Click the **X** to close this window.

Step 6 – identifying dependencies that have been observed in multiple datasets

A dependency observed in any one screen may be a statistical artefact, a context specific dependency, or a false positive resulting from the off-target effects of gene targetting reagents. Those dependencies observed in multiple independent datasets may make more promising candidates as they are less likely to be artefacts or false positive effects. To prioritise these for further validation, CancerGD allows easy filtering of the dependencies observed independently in multiple datasets. To view dependencies that have been associated with the same driver gene in the same tissue type, select '*Yes*' in the '*Multiple Hit*' column toggle. You will see a view resembling the below.

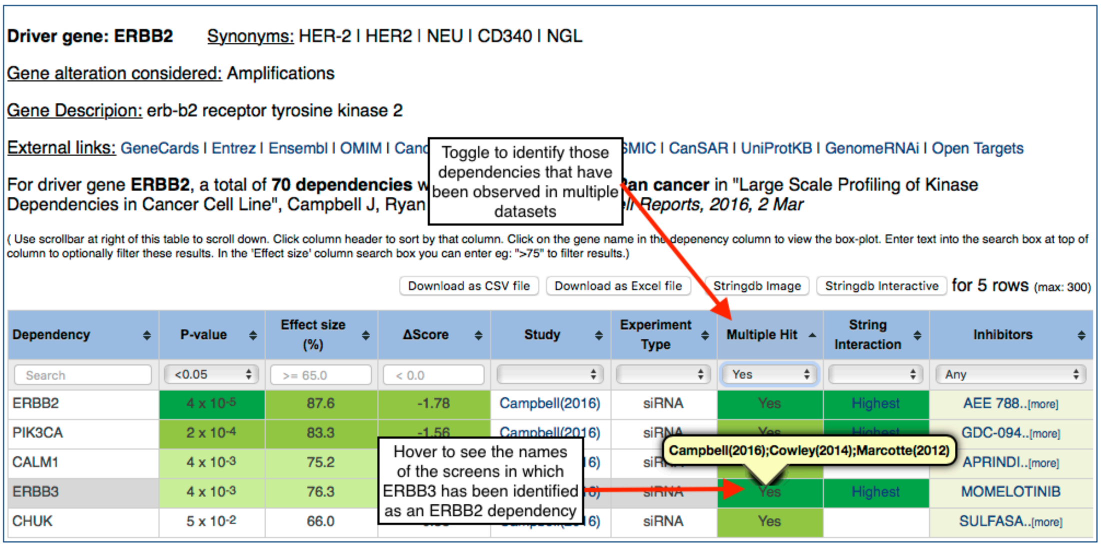

Hover over the *'Yes'* text in the "Multiple Hit" column to see the details of the screens that a specific gene has been identified as a dependency in.

Conclusion

You have now completed a tour of the main www.cancergd.org functionality. Further information is available on the FAQ (http://www.cancergd.org/faq/) page. We welcome feedback through the contact page (http://www.cancergd.org/contact/).
